# Epigenetic age prediction

**DOI:** 10.1111/acel.13452

**Published:** 2021-08-20

**Authors:** Daniel J. Simpson, Tamir Chandra

**Affiliations:** ^1^ MRC Human Genetics Unit MRC Institute of Genetics and Molecular Medicine University of Edinburgh Edinburgh UK

**Keywords:** ageing, composite predictors, epigenetic clocks, minimised clocks, mortality

## Abstract

Advanced age is the main common risk factor for cancer, cardiovascular disease and neurodegeneration. Yet, more is known about the molecular basis of any of these groups of diseases than the changes that accompany ageing itself. Progress in molecular ageing research was slow because the tools predicting whether someone aged slowly or fast (biological age) were unreliable. To understand ageing as a risk factor for disease and to develop interventions, the molecular ageing field needed a quantitative measure; a clock for biological age. Over the past decade, a number of age predictors utilising DNA methylation have been developed, referred to as epigenetic clocks. While they appear to estimate biological age, it remains unclear whether the methylation changes used to train the clocks are a reflection of other underlying cellular or molecular processes, or whether methylation itself is involved in the ageing process. The precise aspects of ageing that the epigenetic clocks capture remain hidden and seem to vary between predictors. Nonetheless, the use of epigenetic clocks has opened the door towards studying biological ageing quantitatively, and new clocks and applications, such as forensics, appear frequently. In this review, we will discuss the range of epigenetic clocks available, their strengths and weaknesses, and their applicability to various scientific queries.

## Graphical abstract

Over the past decade, the repertoire of DNA methylation‐based age predictors, known as epigenetic clocks, has grown. Here, we review four main types of epigenetic clocks that have been developed; human‐array based, reduced, composite and non‐human.

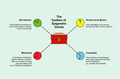

## 1 INTRODUCTION

Ageing is universally accompanied by a decline in physical and cognitive abilities and an increased disease risk. Advanced age is the strongest risk factor common to cardiovascular, neurodegenerative and malignant diseases (Benayoun et al., 2015). At a cellular level, the hallmarks of ageing include genomic instability, telomere attrition, epigenetic alterations, loss of proteostasis, de‐regulated nutrient sensing, mitochondrial dysfunction, cellular senescence, stem cell exhaustion, and altered intercellular communication (reviewed in López‐Otín et al., 2013).

Initial evidence that ageing could be decelerated came from genetic studies of short‐lived non‐vertebrate model organisms (Jia et al., 2004; Kapahi et al., 2004; Kennedy et al., 1995; Klass, 1977; Murphy et al., 2003; Tatar et al., 2001; Tissenbaum & Ruvkun, 1998; Vellai et al., 2003). Nutrient‐sensing pathways were identified as regulators of ageing, such as mechanistic target of rapamycin (mTOR) and insulin/insulin‐like growth factor (IGF) signalling, which can be manipulated to extend lifespan in mammals such as mice (Blüher et al., 2003; Holzenberger et al., 2003; Johnson et al., 2013; Pearson et al., 2008; Weindruch et al., 1986). Other results based on cellular reprogramming showed epigenetic rejuvenation in mice and humans might be possible (Lu et al., 2020; Manukyan & Singh, 2012, 2014; Ocampo et al., 2016; Olova et al., 2019; Sarkar et al., 2020; Singh & Zacouto, 2010). However, to efficiently quantify the effect of these interventions, a biomarker that infers biological age is required, that is a biological indicator that predicts the health and lifespan of an individual better than chronological age (chAge) (Baker & Sprott, 1988).

DNA methylation (DNAm), specifically 5‐methyl‐cytosine (5mC), has emerged as one of the most efficient biomarkers to predict biological age (Benayoun et al., 2015; Jylhävä et al., 2017; Li et al., 2020). In the past decade, a large number of age predictors utilising DNAm have been developed. These DNAm age predictors (more commonly known as epigenetic clocks) are created using CpGs that have tractable changes with age. The majority of these clocks are built using penalised regression models (such as elastic net (Zou & Hastie, 2005) or LASSO (Tibshirani, 1997)), which select a group of CpGs that have a monotonically increasing relationship with age in a given training data (Horvath & Raj, 2018). In other words, key CpGs whose age‐related hyper‐ and hypomethylation correlate with age, are selected and weighted in a linear model. The result is an equation, whereby chronological age can be estimated based on the percentage methylation at these key CpG sites in a given sample.

Epigenetic clocks have become increasingly diverse, with each predictor capturing different aspects of ageing. The expanding repertoire of clocks enable the study of ageing and rejuvenating approaches quantitatively. This review aims to give an overview of the growing toolbox of eAge clocks to inform which approach might best be suited to a scientific question.

## 2 EPIGENETIC CLOCKS PREDICT BIOLOGICAL AGE

Epigenetic clocks have proven themselves to be accurate at predicting chronological age (chAge), which is commonly referred to as DNAm age or epigenetic age (eAge). When epigenetic clocks first emerged, a fundamental question arose; if eAge deviates significantly from chAge, is this difference due to inaccuracies of the clock itself, or caused by biological factors (e.g. genetics, disease status and environment)? In other words, are the clocks able to predict biological age? This difference between eAge and chAge is referred to as age acceleration and can be calculated as the mean absolute deviation (MAD) or median absolute deviation between eAge and chAge (Horvath, 2013), or as the residual from the linear regression between eAge and chAge (Horvath & Raj, 2018). The reported error of epigenetic clocks can depend on how the test/validation data set was curated. For example, if a pooled data set were split into 90% samples for training and 10% for validation, then the clock would perform better on the validation data set than when applied to a completely independent/external data set. In the various tables surmising epigenetic clocks throughout this review, we will clarify how each clock is validated.

For many of the eAge predictors, age acceleration is associated with a number of age‐related diseases and conditions. For example, patients with Down's syndrome (Horvath, Garagnani, et al., 2015), HIV (Horvath & Levine, 2015), obesity (Horvath et al., 2014), Huntington's disease (Horvath, Langfelder, et al., 2016), Werner syndrome (Maierhofer et al., 2017) and Sotos syndrome (Martin‐Herranz et al., 2019) tend to exhibit increased age acceleration. eAge acceleration has also been associated with physical and cognitive fitness (Breitling et al., 2016; Marioni, Shah, McRae, Ritchie, et al., 2015; Quach et al., 2017) and neuropathy (Levine et al., 2015; Lu et al., 2017) (for comprehensive lists of age acceleration‐associated conditions, please refer to Horvath & Raj, 2018 and Declerck & Berghe, 2018). Variation in epigenetic ageing rates between individuals has been shown to significantly depend on sex and race/ethnicity (Horvath, Gurven, et al., 2016; McCartney et al., 2019). Vitamin D‐sufficient individuals have a lower eAge acceleration and longer leukocyte telomere length (LTL) (Chen et al., 2019; Vetter et al., 2020). Smoking has been associated with an increase in eAge in airway cells and lung tissue (4.9 and 4.3 years, respectively) (Wu et al., 2019), and smoking during pregnancy might have an effect on eAge in offspring (Simpkin et al., 2016). The number of studies associating eAge acceleration with diseases, phenotypes and environmental interventions that appear to affect ageing, emphasises eAge as a candidate metric for biological age (Wang et al., 2017). However, some studies have shown no correlation between eAge acceleration and certain diseases or environmental factors, such as type II diabetes (Grant et al., 2017; Horvath, Gurven, et al., 2016), heroin use (Kozlenkov et al., 2017) or depression (Starnawska et al., 2019). Why eAge acceleration tracks with certain age‐related disorders and not others are still not well understood. Most epigenetic clocks described in the following section used Illumina DNAm array‐based technology and are summarised in Table 1.

**TABLE 1:** Epigenetic clocks based on Illumina human DNA methylation arrays

Clock	No. CpGs	Error (Years)	Generation of error estimate (type of validation data set used)	No. of samples in training	Method used to find age‐associated CpGs	Age range of training	Cell types/Tissue used for training	Additional functional tissues/Cells	Reference
Bocklandt	88	5.2	Leave‐one‐out	68 (34 twin pairs)	CpGs with *q* < 0.05 & absolute corr >0.57 with age	21–55	Saliva	‐	Bocklandt et al. (2011)
Koch & Wagner	5	11	Independent validation data set	150	Pavlidis Template Matching	16–72	Fibroblasts, keratinocytes, epithelial, peripheral blood	Saliva, breast organoid	Koch and Wagner (2011)
Passage Number	6	‐	‐	‐	Pavlidis Template Matching	‐	Fibroblasts, mesenchymal stem cells	‐	Koch et al. (2012)
Horvath (Pan‐Tissue)	353	Median Absolute Deviance 3.6	Independent validation data set	3931	Elastic net regression	0–100	51 different tissues/cell types including blood, brain, muscle	‐	Horvath (2013)
Skin & Blood (S&B)	391	No overall MAD for all tissues /cell types	Independent validation data set	896	Elastic net regression	0–94	Fibroblasts, keratinocytes, buccal cells, endothelial cells, lymphoblastoid, skin, blood, saliva	Brain, neurons, glia, liver, bone	Horvath et al. (2018)
Zhang (Elastic Net)	514	RMSE 2.04	Independent validation data set	13,661	Elastic net regression	2–104	Whole blood, saliva	Breast, liver, adipose, muscle, endometrium	Zhang, Vallerga, et al. (2019)
Zhang (BLUP)	319,607	RMSE ~2.04	Independent validation data set	13,661	Best linear unbiased prediction	2–104	Whole blood, saliva	‐	Zhang, Vallerga, et al. (2019)
Hannum	71	RMSE 4.9	Independent validation data set	482	FDR to filter significant CpGs then elastic net	19–101	Whole blood	‐	Hannum et al. (2013)
Weidner (102 CpG)	102	3.3	Independent validation data set	575	CpGs selected by pearson corr (*r* > 0.85 or *r* < −0.85)	0–78	Whole blood	‐	Weidner et al. (2014)
Weidner (99 CpG)	99	4.1	Independent validation data set	656	CpGs derived from 102 previous CpGs in Weidner et al. (2014)	19–101	Whole blood	‐	Weidner et al. (2014)
Weidner/Lin (3 CpG)	3	7.6	Independent validation data set	656	Three CpGs selected from 102 previous CpGs, recursive feature elimination	19–101	Whole blood	‐	Weidner et al. (2014), Lin et al. (2016)
Boroni Skin	2,266	RMSE 4.98	Random segregation of validation data set from training	249	Elastic net regression	18–95	Dermis, epidermis, whole skin	‐	Boroni et al. (2020)
Pediatric‐Buccal‐ Epigenetic (PedBE)	94	0.35	Independent validation data set	1,032	Elastic net regression	0–19.5	Buccal epithelial cells	‐	McEwen et al. (2019)

Note

Age‐associated CpGs are selected and weighted in a linear model, resulting in epigenetic age predictors (epigenetic clocks). Error (years) is based on mean absolute deviation (MAD) unless otherwise stated.

## 3 DNAM ARRAY‐BASED EPIGENETIC CLOCKS

### 3.1 Early epigenetic age predictors

The first epigenetic clocks incorporated relatively few CpG sites and samples in their training data sets, in comparison with later clocks. Bocklandt et al., for example created a clock from 68 samples (34 twin pairs) that predicts age in saliva with an average accuracy of 5.2 years (Bocklandt et al., 2011). Koch and Wagner used five CpG sites and predicted age in multiple cell types, but with lower accuracy than the Bocklandt clock (MAD = 11 years) (Koch & Wagner, 2011). The same laboratory produced a six CpG clock that could track passage number in fibroblast cell cultures, regardless of original donor age (Koch et al., 2012). After these initial studies, epigenetic clocks grew in complexity in terms of number of samples, tissues and CpGs implemented.

### 3.2 Multi‐tissue age predictors

The first multi‐tissue age predictor (referred to as the Horvath or Pan‐Tissue clock) utilised 353 CpGs and has a mean error of 3.6 years, which at that time was unprecedented for any biomarker/age predictor (Horvath, 2013). The training data set used to construct the clock comprised of 8000 samples from 82 studies, including 51 healthy tissues and cell types. The size of the training data was a step‐change in clock design. Hence, the Horvath clock gained popularity in the scientific community since it can predict age in multiple tissues using a relatively small number of CpGs (compared to the rest of the epigenome) and revealed that tissues may age at different rates. For example, brain tissue appears to age slower relative to other tissues in the body, according to the Horvath clock (Horvath, 2013; Horvath, Mah, et al., 2015).

The association between age acceleration and health/disease status was first shown with the Horvath clock in obesity (Horvath et al., 2014) and has since become an established tool to assess biological age (Horvath et al., 2014; Horvath, Garagnani, et al., 2015; Horvath, Langfelder, et al., 2016; Horvath & Levine, 2015; Maierhofer et al., 2017; Martin‐Herranz et al., 2019). The Horvath clock has shown some limitations with particular tissues and age‐associated disease conditions. One of the most severe premature ageing syndromes, Hutchinson‐Gilford Progeria Syndrome (HGPS), did not exhibit age acceleration according to the Horvath clock (Horvath, 2013). Children with multifocal developmental dysfunctions (syndrome X), who appear to age slower, do not decelerate in eAge (Walker et al., 2015). However, being rare genetic disorders, both studies were limited in the number of individuals tested.

The Horvath clock does not work reliably on cultured cells, particularly fibroblasts (Horvath, 2013; Horvath et al., 2018, 2019). Replicative senescence in primary fibroblasts is a widely used model system in cellular ageing (Chandra & Kirschner, 2016; Hayflick, 1965; Hayflick & Moorhead, 1961). More recently, Horvath et al. developed an epigenetic clock that predicts the age of human fibroblasts, keratinocytes, buccal cells, endothelial cells, lymphoblastoid cells, skin, blood and saliva samples, better than the original Horvath clock (Horvath et al., 2018). This clock, known as the skin and blood (S&B) clock, is able to predict both *in vivo* and *in vitro* tissues accurately (Horvath et al., 2018, 2019). The S&B clock also detected a modest, but significant age acceleration in HGPS samples (Horvath et al., 2018).

The Zhang clock, while primarily trained to work on blood, is able to predict the ages of breast, liver, adipose and muscle tissue as accurately as the Horvath clock (Zhang, Vallerga, et al., 2019). This clock also outperformed both the Horvath and Hannum clocks in predicting blood age. It is set apart by the size of its training data with over 13,000 samples.

### 3.3 Tissue‐specific age predictors

A number of CpG clocks have been developed for single tissues, aiming at an increased accuracy for a given cell type or specialised applications. Multiple clocks have been developed for blood, the first of which was the Hannum clock (Hannum et al., 2013; Horvath & Raj, 2018; Weidner et al., 2014; Zhang et al., 2017; Zhang, Kamath, et al., 2019; Zhang, Vallerga, et al., 2019). A later study found 102 CpG sites that can predict age in blood, 99 of which were adapted for a separate clock that works on the Illumina 450 K array (Weidner et al., 2014). It was demonstrated that three of the 102 CpGs alone (selected by recursive feature elimination) can predict age in arrays and pyrosequenced samples (Lin et al., 2016; Weidner et al., 2014). A minimal approach, such as this that uses as few CpGs as possible, is a sought after technique for affordable use in clinical and forensic fields (see “Minimised CpG Clocks”).

A study by Boroni et al. has produced an accurate skin age predictor, based on 2266 CpGs (one of the largest number of CpGs used to create an eAge clock) selected by elastic net regression (Boroni et al., 2020). It was trained on dermis, epidermis and whole skin biopsies (40, 99 and 110 samples, respectively) and had a root mean squared error (RMSE) of 4.98 when tested on an external validation data set of whole skin biopsies (by comparison, the Horavth and S&B clocks had RMSEs of 15.74 and 7.64, respectively) (Boroni et al., 2020).

Inaccuracies in epigenetic clocks are apparent when predicting the age of younger individuals (under 20 years old) (Simpkin et al., 2016). This might be due to insufficient numbers of young individuals in training data sets, or due to the linear models used to construct the epigenetic clocks (see “Inaccuracies and Tick‐Rate of Epigenetic Clocks”). The Pediatric‐Buccal‐Epigenetic (PedBE) clock was developed for use in 0‐ to 20‐year‐olds and trained on a large number of buccal swab samples (1,032, aged 0–19.5 years old) (McEwen et al., 2019). This clock performs well (MAD = 0.35 years) and is an example of how the accuracy of epigenetic clocks can be improved not only by targeting specific tissues, but also specific age groups.

### 3.4 Minimised CpG clocks

Most of the epigenetic clocks mentioned above rely on Illumina Infinium arrays. The price of these arrays limits the applicability of eAge technology in drug discovery. Drug‐discovery pipelines require the multiplexing of thousands of samples, but not necessarily the accuracy of the arrays. Here, we will discuss clocks relying on fewer CpGs (minimised clocks), which have the potential to be upscaled or run at a lower sample cost. The forensics field has developed multiple minimised clocks using strong age‐associated CpGs (such as ELOVL2 and FHL2 (Garagnani et al., 2012; Bacalini et al., 2017)) and is designed for common tissues found at crime scenes, such as blood, saliva, buccal swabs and semen (Table 2).

**TABLE 2:** Minimised CpG epigenetic clocks

Clock	Sequencing	No. CpGs	Error (Years)	Generation of error estimate (type of validation data set)	No. of samples in training	Method used to find age‐associated CpGs	Age range of training	Cell types/Tissue used for training	Reference
Weidner 3 CpG	Bisulphite pyrosequencing	3	4.5	Independent validation data set	82	3 CpGs selected from 102 previous CpGs by recursive feature elimination	0–78	Whole blood	Weidner et al. (2014)
Eipel Buccal	Bisulphite pyrosequencing	5	5.1	Independent validation data set	55	3 CpGs from Weidner et al. (2014), plus two additional buccal‐specific CpGs	1–85	Saliva	Eipel et al. (2016)
Zbieć‐Piekarska (ZP) Clock	Bisulphite pyrosequencing	5	3.9	Independent validation data set	420	8 CpGs from Hannum et al. (2013) then multivariate linear regression	2–75	Peripheral blood	Zbieć‐Piekarska et al. (2015)
Cho Model 2	Bisulphite pyrosequencing	5	4.2	Independent validation data set	100	Similar CpGs to ZP clock (same associated genes but different CpGs), trained in multivariate regression model	20–74	Whole blood	Cho et al. (2017)
Jung‐Blood	SNaPShot	5	3.5	Independent validation data set	100	CpGs used by Cho Model 2 (with different Clorf132 CpG) retrained in multivariate linear model	~19–70	Whole blood	Jung et al. (2019)
Jung‐Saliva	SNaPShot	5	3.6	Independent validation data set	100	""	~19–70	Saliva	Jung et al. (2019)
Jung‐Buccal Swab	SNaPShot	5	4.3	Independent validation data set	100	""	~19–70	Buccal epithelial cells	Jung et al. (2019)
Jung‐Mixed Tissue	SNaPShot	5	3.8	Independent validation data set	300	""	~19–70	Whole blood, saliva, buccal epithelial cells	Jung et al. (2019)
Dias‐Deceased Clock	Bisulphite PCR	5	8.8	Independent validation data set	51 (Deceased)	PCR of CpGs from previous studies, trained in multivariate linear model	24–86	Blood	Dias, Cordeiro, Corte Real, et al. (2020a)
Dias‐Multi‐Locus Model	Bisulphite PCR	4	5.4	Random segregation of validation data set from training	53	Using CpGs/regions previously used in Dias, Cordeiro, Corte Real, et al. (2020a), trained in multivariate linear model	1–95	Peripheral blood	Dias, Cunha, et al. (2020)
Dias‐Blood (5 CpG)	SNaPShot	5	4.3	Random segregation of validation data set from training	59	Same CpGs used by Jung et al. (2019), retrained in multivariate linear model	1–94	Peripheral blood	Dias, Cordeiro, Pereira, et al. (2020)
Dias‐Blood (3 CpG)	SNaPShot	3	4.8	Random segregation of validation data set from training	59	3 of 5 CpGs used by Jung et al. (2019) were retrained in multivariate linear model	1–94	Peripheral blood	Dias, Cordeiro, Pereira, et al. (2020)

Note

Epigenetic clocks created using a low number of CpGs (typically under 10), usually from preselected CpGs/regions known to have high age correlation. Error is based on mean absolute deviation (MAD).

Minimised clocks use a variety of technologies such as the Qiagen platform for pyrosequencing (referred to as pyrosequencing from here), which is more cost‐effective for profiling the methylation of select CpGs. The Weidner 3 CpG clock (see “Tissue‐Specific Age Predictors”) for example, can predict age in blood samples using pyrosequencing (Weidner et al., 2014), but over‐predicts age in saliva (a common source of DNA at crime scenes) by 14.6 years on average (Eipel et al., 2016). When adapted for saliva by adding two additional buccal‐specific age‐associated CpGs, eAge prediction was improved (Eipel et al., 2016).

Pyrosequencing had its own limitations (e.g. multiplexing; allowing a large number of samples and CpGs to be pooled and sequenced in a single run); however, new approaches increasing multiplex capabilities in pyrosequencing are emerging (Fleckhaus & Schneider, 2020). Another assay, termed SNaPshot, can multiplex 10 CpG sites (Thermo Fisher, 2020) and is used for many minimised epigenetic clocks.

The use of minimised clocks in forensics is just developing and for most clocks, cross‐validation is missing (Cho et al., 2017). However, the clock by Zbiec´‐Piekarska et al. has been validated and adapted in other studies. It is based on 5 out of 8 CpGs previously identified by Hannum et al. as showing the strongest age association (Hannum et al., 2013; Zbiec´‐Piekarska et al., 2015) and has a standard error of 4.5 years and an MAD of 3.9 years. The genes associated with these CpGs are *ELOVL2*, *C1orf132*, *TRIM59*, *KLF14* and *FHL2*. A clock based on five CpGs (located near the same previous genes) was created by Cho et al. and has been validated in multiple tissues (Cho et al., 2017; Dias, Cordeiro, Pereira, et al., 2020; Jung et al., 2019). These CpGs not only operate adequately with SNaP‐shot assays as tissue‐specific age predictors, but also as a multi‐tissue age predictor for common forensic tissues (blood, saliva and buccal swab) (Jung et al., 2019). Three of the CpG sites (near *ELOVL2*, *FHL2* and *C1orf132*) have also proven sufficient to predict age efficiently (Dias, Cordeiro, Pereira, et al., 2020). These studies have demonstrated the versatility and accuracy predictors based on a few select CpGs can have and might be good candidates to increase the scale of eAge prediction.

## 4 COMPOSITE EPIGENETIC CLOCKS AS PREDICTORS OF MORBIDITY AND MORTALITY

Epigenetic clocks have proven capable of estimating not only chAge, but also time‐to‐death. Marioni et al. first showed that the higher difference between eAge and chAge, the greater the risk of all‐cause mortality (mortality independent of health status, known genetic factors, and lifestyle factors) (Marioni, Shah, McRae, Chen et al., 2015). This finding was further validated in other studies (Chen et al., 2016; Christiansen et al., 2016). Positive age acceleration was also shown to predict cause‐specific mortality in cancer and cardiovascular disease (Perna et al., 2016). These mortality associations were found using clocks that were not designed to directly predict mortality. Various composite approaches have been developed, whereby CpGs that correlate with metrics of physiological or cellular ageing (e.g. cholesterol or protein abundance) are used to construct a clock to predict age (Table 3). These clocks were built with the potential of capturing more of age‐relevant biology than clocks trained on chAge alone.

**TABLE 3:** Composite and mortality epigenetic clocks

Clock	No. CpGs	Method used to obtain CpGs	No. of samples in training	Reference
PhenoAge	513	Elastic net	9,926	Levine et al. (2018)
GrimAge	1,113	Elastic net	1,731	Lu, Quach, et al. (2019)
Zhang Mortality Clock	10	LASSO Cox regression	548	Zhang, Wilson, et al. (2017)
DunedinPoAm	46	Elastic net	810	Belsky et al. (2020)
Telomere Clock	140	Elastic net	2,256	Lu, Seeboth, et al. (2019)

Note

All clocks in this table are composite clocks, i.e. CpGs that correlate with physiological or cellular ageing are used to create a biological age predictor (except the Zhang Mortality Clock, where mortality data were directly regressed on DNAm).

The first composite biomarker age predictor (created independent of DNAm data) was based on 23 years of mortality data (Levine, 2013). This predictor incorporated ten biomarkers (e.g. C‐reactive protein, glycated haemoglobin, systolic blood pressure, total cholesterol) that significantly correlated with age (Levine, 2013). Using a similar process, Levine et al. combined chAge plus nine other biomarkers. The resulting phenotypic clock was regressed on DNA methylation data using elastic net regression, resulting in 513 CpGs forming the DNAm PhenoAge clock (Levine et al., 2018). This clock predicts all‐cause mortality, cancer, healthspan, physical functioning and Alzheimer's disease more accurately than previous age predictors (Levine et al., 2018).

The GrimAge clock developed by Lu et al. uses the methylation of CpGs associated with smoking (pack‐years) and levels of 7 plasma proteins previously associated with mortality (Ignjatovic et al., 2011; Ridker et al., 2003), as surrogates for physiological risk factors (Lu, Quach, et al., 2019). The age acceleration of GrimAge was not only found to be associated with age‐related conditions and lifestyle factors, but outperformed previous attempts at predicting time‐to‐death, time‐to‐coronary heart disease and time‐to‐cancer (Lu, Quach, et al., 2019). A significant association has been shown between GrimAge acceleration and lifelong trauma, but not childhood trauma (Katrinli et al., 2020), which is consistent with other studies showing Hannum (Wolf et al., 2016) and Horvath (Yang et al., 2020) clock age accelerations in post‐traumatic stress disorder. GrimAge acceleration is also significantly associated with cortical atrophy (Katrinli et al., 2020), shorter pregnancy periods and lower birthweight (Ross et al., 2020).

Zhang et al. created a mortality‐specific predictor, where they performed an epigenome‐wide association study (EWAS) on a cohort with up to 14 years follow‐up data. 58 CpGs were found that correlate with all‐cause mortality, from which a predictor was constructed using only ten of the CpGs (Zhang, Wilson, et al., 2017). 48 of the CpGs identified had been associated with smoking, alcohol consumption, diabetes and cancer, some of which were also found in previous EWAS studies (Al Muftah et al., 2016; Chambers et al., 2015; Gao et al., 2015; Nilsson et al., 2014; Teschendorff et al., 2015; Travers et al., 2013; Zhang, Wilson, et al., 2017).

A DNAm telomere length (DNAmTL) estimator was created by Lu, Seeboth, et al. (2019), where leukocyte telomere length (LTL) was regressed against blood methylation data. This resulted in 140 LTL‐associated CpGs forming the DNAmTL estimator (Lu, Seeboth, et al., 2019). Not only does DNAmTL predict LTL accurately, but it also demonstrates stronger predictive power of lifespan, time‐to‐coronary heart disease, time‐to‐congestive heart failure and smoking history compared to normal LTL.

Variability in early‐life environmental exposures has been proposed as one of the main confounders of mortality clocks (Bell et al., 2019; Hillary et al., 2020). Belsky et al. addressed this directly, by analysing rate of change of 18 blood‐chemistry and organ‐system‐function in a cohort with the same birth year and birth place (Belsky et al., 2015; Hillary et al., 2020). Termed “Pace‐of‐Ageing” (PoA), this measure formed the basis of the DunedinPoAm clock, a proxy approach with PoA regressed on DNAm (Belsky et al., 2020). In other words, DunedinPoAm aims to provide the rate of biological ageing at a single‐time‐point of a person (Belsky et al., 2020).

The Marioni laboratory compared the performance of six of the epigenetic age/mortality predictors mentioned (Horvath, Hannum, PhenoAge, GrimAge, DNAmTL and Duned‐ inPoAm) in terms of lifespan and disease prediction, on the Generation Scotland cohort (Hillary et al., 2020). GrimAge overall had the best performance; it predicted the prevalence of chronic obstructive pulmonary disease (COPD) and the incidence of multiple diseases, including COPD, type 2 diabetes and cardiovascular disease. GrimAge also outperformed other clocks for predicted death in terms of all‐cause mortality, after adjustment for lifestyle risk factors. Another recent study also showed GrimAge outperforms Horvath, Hannum and PhenoAge clocks at predicting all‐cause mortality and age‐related clinical phenotypes (McCrory et al., 2021). However, DunedinPoAm did reveal faster rates of biological ageing associated with lung cancer and COPD. PhenoAge and DNAmTL also showed associations with disease incidence for type 2 diabetes and ischaemic heart disease, respectively. Hence, composite clocks can use DNAm to predict non‐DNAm traits, which in turn can be used as additional variables to accurately predict biological age, disease status and mortality.

## 5 UNDERLYING MECHANISM OF THE EPIGENETIC CLOCK

The Horvath clock is the most widely used clock for its accuracy, versatility and the accumulated knowledge we have of its behaviour from previous studies. It also gained the attention of the scientific community due to the fact that age can be predicted in multiple tissues using a relatively small number of CpGs (compared to the rest of the epigenome; Horvath, 2013). The fact that such a clock can be constructed provokes the question, is there a functional significance that correlates these CpGs with age in multiple tissues? If ageing is a phenomena that we are “programmed” to undergo, then are these CpGs an integral part of that machinery? To understand the nature of eAge/epigenetic clocks, we must understand the aspects of physiological ageing they capture, the CpGs that constitute these clocks, and any causative relationships with ageing.

### 5.1 Inaccuracies and tick‐rate of epigenetic clocks

As with the Horvath clock, most clocks that followed after were also built on penalised linear regression models. However, are there intrinsic inaccuracies in the Horvath clock, and the approach used to construct epigenetic clocks? El Khoury et al. analysed previously published DNAm data sets and found that both the Horvath and Hannum clocks systematically underestimate the age of older individuals (El Khoury et al., 2019). If age acceleration is dependent on chAge itself, biological interpretation of age acceleration at very old age becomes difficult. Centenarian peripheral blood mononuclear cells are predicted 8.6 years younger than their chAge, according to the Horvath clock (Horvath, Pirazzini, et al., 2015). Similar findings were also found in analysis of cerebellum tissue from supercentenarians (Horvath, Mah, et al., 2015). The interpretation has been that the younger age predicted for centenarians reflects survival bias, where the lower biological age enabled the centenarians to live long. However, with the clock possibly underpredicting older age systematically, this assumption might need to be reexamined. Alternatively, this discrepancy might be the result of a regression to the mean effect, where very high values (eAges) are underestimated by regression models.

While negative age acceleration (eAge predicted lower than chAge) was highest in the cerebellum, this underestimation was also observed in other tissues (including blood) from multiple data sets (El Khoury et al., 2019; Marioni et al., 2019; Martin‐Herranz et al., 2019). It was also found that when accounting for age as a cofactor, the correlation between age acceleration and amyloid plaque load in brain tissue is attenuated (El Khoury et al., 2019), which is inconsistent with previous findings (Levine et al., 2015). It is possible that 5‐hydroxymethyl cytosine (5hmC, an epigenetic modification more prevalent in brain tissue and indistinguishable from 5mC after bisulphite conversion) could cause age prediction offset in brain tissue (El Khoury et al., 2019; Lunnon et al., 2016). However, 5hmC is not prevalent in blood and therefore does not explain the negative age acceleration in blood detected by Marioni et al. (2019), El Khoury et al. (2019). These alterations in predictive accuracy of the clock in older individuals could be due to intrinsic changes in the rate of biological ageing during certain time points. The rate of change, or “tick” rate, was explored earlier in the Horvath clock study (Horvath, 2013). By looking at the weighted averages of the 353 CpGs compared with chAge, the tick rate was exponential between 0 and 20 years old, after which it continued linearly. As such, the Horvath clock applies a logarithmic transformation to ages <20 years, while the linear model is unaltered for ages >20 years (Horvath, 2013; Snir et al., 2019). The study suggested that a higher organismal growth and cell division rate at early age might explain the initial acceleration in ageing (Horvath, 2013). A later study found a faster eAge tick rate during puberty in girls (Binder et al., 2018). However, no decrease in the tick rate of older subjects was observed (Horvath, 2013), which could be due to a lack of older individuals in the training data set used to construct the Horvath clock (El Khoury et al., 2019). Differences in tick rate could also be sex‐specific. The Horvath, Hannum, and Zbiec´‐Piekarska clocks show slightly faster ageing in men than women (Bergsma & Rogaeva, 2020).

A recent study found that simple multiple linear regression outperforms more involved machine learning techniques (Lau & Fung, 2020). However, if there is indeed a non‐linear progression of age acceleration, then other models might be worth exploring to predict eAge. Deep learning and support vector regression are other alternatives to penalised linear regression that have been used (Aliferi et al., 2018; Galkin et al., 2020, 2021; Levy et al., 2020; Xu et al., 2015). The epigenetic pacemaker (EPM) is another algorithm where predicted age follows a logarithmic trend (Snir et al., 2016, 2019). Whether EPM or other non‐linear models predict eAge in centenarians more accurately has not been determined.

### 5.2 What aspects of physiological ageing does eAge capture?

eAge acceleration (eAge higher than chAge) or deceleration (eAge lower than chAge) is reflected in many diseases (e.g. Down syndrome) and environmental factors (e.g. smoking) that appear to increase or decrease ageing at a physiological level (Chen et al., 2016; Higgins‐Chen et al., 2020; Horvath et al., 2014; Horvath, Langfelder, et al., 2016; Horvath et al., 2018; Horvath & Levine, 2015; Horvath, 2015, 2015c; Maierhofer et al., 2017; Marioni, Shah, McRae, Chen, et al., 2015; Martin‐Herranz et al., 2019; Simpkin et al., 2016; Wu et al., 2019). What remains unclear is whether eAge reflects or measures known physiological/cellular ageing phenomena (e.g. telomere length, senescence).

Consistent eAge prediction between tissues of an individual suggests that eAge is not a measure of cellular proliferation, since different tissues have variable proliferation rates (Horvath, 2013; Horvath et al., 2019; Horvath, Mah, et al., 2015). Indeed, multiple studies have shown that while eAge changes with cell passage number, the Horvath age predictor does not rely on cell division since it can track eAge in non‐proliferative tissues (e.g. neuronal cells; Horvath, 2013; Yang et al., 2016; Horvath et al., 2019). A mitotic clock (EpiTOC) has been developed specifically to track cell divisions, and acceleration of this clock correlates with cancer status (Yang et al., 2016). It would be intuitive to assume that eAge reflects other known aspects of ageing such as senescence, since an increase in senescence cells is considered a hallmark of ageing (Horvath et al., 2019; López‐Otín et al., 2013). However, this has not been shown; instead, both replicative and damage‐induced senescence do not correlate with increased eAge in vitro (Horvath et al., 2019; Lowe et al., 2016). It is possible that the accumulation of senescent cells in tissues with age remains proportionally low and that it is the effect on surrounding cells that is registered in eAge. Human telomerase reverse transcriptase (hTERT) expressing cells continue to epigenetically age despite never being able to enter replicative senescence (Kabacik et al., 2018). Leukocyte telomere length (LTL) erosion is one of the first biological phenomena that showed potential as biomarkers of ageing (Frenck et al., 1998; Harley et al., 1990; Hastie et al., 1990; Lindsey et al., 1991) and could be a physiological sign of ageing that correlates with eAge. However, like cellular proliferation and senescence, multiple studies have shown that eAge has no association with telomere length (Cypris et al., 2020; Horvath et al., 2019; Kabacik et al., 2018; Lowe et al., 2016; Marioni et al., 2016).

A plausible alternative is that eAge is governed by cellular differentiation. As stem cells divide during development, they differentiate into different cell types as the embryo matures, which could be reflected by changes in eAge. One study tested the influence of tissue identity on eAge by growing keratinocytes in a media that encourages differentiation. No increase of eAge was observed in the differentiating keratinocytes compared to the non‐differentiating, proliferating keratinocytes (Horvath et al., 2019). A separate study transdifferentiated fibroblasts to neurons using miRNAs. The reprogrammed neurons not only had a similar eAge as the donor fibroblasts but also similar telomere length, oxidative stress and DNA damage (Huh et al., 2016), suggesting that direct reprogramming had no effect on eAge.

It has been hypothesised that eAge‐related changes are reflected in intracellular alterations and changes in cell composition in a subset of cells termed “clock cells” (Horvath & Raj, 2018). eAge might therefore capture the loss of somatic cells in some tissues (Horvath & Raj, 2018) or the loss of stem cells, which do decline during ageing (Hernando‐Herraez et al., 2019). A caveat is that eAge can be captured in neuronal cells, which are terminally differentiated cells and lack a stem cell pool (Horvath, 2013; Horvath, Mah, et al., 2015; Horvath & Raj, 2018).

It also possible that eAge measures aspects of age‐related epigenetic drift or deregulation (Yu et al., 2020). Demethylation can occur in either a passive manner (e.g. via inhibition of DNMT1 during cell replication; Wolffe et al., 1999; Mayer et al., 2000), or actively via methyl‐CpG binding domain protein 4 (MBD4; Hendrich et al., 1999) or TET enzymes (Ichiyama et al., 2015; Jin et al., 2014). However, there is little evidence to suggest that active processes, such as TET, directly demethylate with age and affect eAge prediction (Wallace, 2014; Yu et al., 2020; Zhang et al., 2016). In addition, eAge can be measured in nonproliferating tissues (Horvath, 2013; Horvath et al., 2019; Yang et al., 2016) meaning passive demethyation is an unlikely mechanism. It is possible that actively dividing tissues accumulate somatic mutations in DNA methylation machinery during ageing, resulting in epigenetic drift observed as aberrant eAge prediction (Robertson et al., 2019).

The precise aspects of physiological ageing that eAge captures remain to be discovered, but further investigations into genes associated with eAge/clock CpGs and associations with other ageing biomarkers may disclose clues to the true nature of eAge.

### 5.3 Causality of clock CpGs in ageing

DNAm became apparent as a potential biomarker of ageing with the discovery of strong age‐associated CpGs, such as those in the CpG islands of ELOVL2, FHL2 and PENK1 (Bacalini et al., 2017; Garagnani et al., 2012). ELOVL2 is a strong biomarker for ageing in multiple tissues in both human and mouse (Bacalini et al., 2017; Chen et al., 2020; Garagnani et al., 2012; Hannum et al., 2013; Slieker et al., 2018). The CpGs neighbouring ELOVL2 strongly hypermethylate with age (Garagnani et al., 2012) and have been used in multiple forensic clocks (see “Minimised CpG Clocks”). ELOVL2 is an enzyme involved in elongation of long‐chain polyunsaturated fatty acids, and also in the production of docosahexaenoic acid (DHA). DHA is the main polyunsaturated fatty acid in the retina and brain, and is necessary for healthy retinal function. Chen et al. showed that the *Elovl2* promoter is more highly methylated in the retina of aged mice and that demethylation of this site recovers age‐related decline in visual function via increased expression of *Elovl2* (Chen et al., 2020). This is one of few studies to test a causal link of age‐associated CpGs with phenotypic ageing.

### 5.4 Transcriptional associations with eAge

One approach to functionally annotating CpGs is to analyse gene expression changes that correlate with the methylation of age‐associated CpGs. In the Horvath clock, the 193 CpGs (out of 353) that hypermethylate with age are more likely to be located in poised (bivalent) promoters. The 160 (out of 353) CpGs that hypomethylate with age are more likely to be in either weak promoters or strong enhancer regions (Horvath, 2013). However, linking the activity of age‐related CpGs with specific gene expression has proven difficult (Horvath & Raj, 2018; Jung & Pfeifer, 2015; Yin et al., 2017; Zheng et al., 2016). The most likely reason is that many age‐associated CpGs might not be related to gene expression. Another reason might be that the epigenetic state of cells in any given tissue is heterogeneous, making associations between methylation and gene expression difficult to find. Dual transcriptomic and epigenetic sequencing at a single cell level could help to establish a functional link between the two (Horvath & Raj, 2018) (Angermueller et al., 2016). A recent study by Hernando‐Herraez et al. used scMT‐seq to assess ageing in mouse muscle stem cells (MuSCs). They isolated young and old quiescent MuSCs and determined that epigenetic drift (specifically, stochastic methylation heterogeneity at promoters) is associated with age‐associated transcriptional heterogeneity (Hernando‐Herraez et al., 2019). They also predicted eAge by aggregating single cells by individual (two young and two old mice, with 35 cells per individual). Their age predictor performed accurately on the young MuSCs; however, their old MuSCs had a similar eAge to the young samples (~10 weeks, while the chAge of the old MuSCs were ~100 weeks). To compensate for this error, they estimated eAge using different combinations of cells and permutations, by removing 5% of cells and calculating eAge of the subsequent sample. The old MuSCs were still ~90 weeks lower than the chronological age (Hernando‐Herraez et al., 2019).

### 5.5 Genetic variants associated with eAge

A genome‐wide association study (GWAS) is a method that could reveal genes that regulate eAge by finding genetic polymorphisms that correlate with eAge. A GWAS of cerebellum tissue found variants near an mTOR complex 2 gene (MLST8) and in an RNA‐helicase gene (DHX57) that are associated with age acceleration. Many genes associated with cerebellar age acceleration also had overlap with neurodegenerative conditions such as Alzheimer's disease (Lu et al., 2016). Another GWAS revealed that one of the loci associated with intrinsic eAge acceleration (IEAA, which adjusts for both chAge and blood cell counts; Horvath, Gurven, et al., 2016; Quach et al., 2017) co‐locates with hTERT (Lu et al., 2018). Variants of hTERT were found that associated with both IEAA and longer telomeres. Moreover, it was showed *in vitro* that higher hTERT expression (which is normally associated with cellular longevity) appears to cause a linear increases of eAge. By comparison, control cells passaged with no hTERT experienced an initial increase in eAge after 33 days in culture that eventually plateaued. These findings further enforce that eAge is not governed by cell division, replicative senescence or telomere length per se (Cypris et al., 2020; Horvath et al., 2019; Kabacik et al., 2018; Lowe et al., 2016; Marioni et al., 2016), since short telomeres are indicative of high proliferation and triggers replicative senescence. This paradoxical result could explain previous observations where during embryonic development and early postnatal life, the rate of epigenetic ageing is more rapid (Hiyama & Hiyama, 2007; Lu et al., 2018; Simpkin et al., 2016, 2017). These are periods of fast organismal growth coupled with high hTERT expression and cell division, which in turn would result in a higher eAge predicition.

Another approach to identify eAge‐associated genetic variants involves screening for developmental disorders that cause an acceleration or deceleration of eAge. This was conducted by Martin‐Herranz et al., who screened 367 genetic disorders, and found that Sotos syndrome significantly accelerated eAge (Martin‐Herranz et al., 2019). Sotos syndrome is caused by a loss‐of‐function mutation in *NSD1*, which encodes a histone H3 lysine 36 (H3K36) methyltransferase (Choufani et al., 2015; Kurotaki et al., 2002). Methylated H3K36 recruits DNMT3A/B and promotes methylation of surrounding regions. The authors hypothesised that H3K36 methylation machinery might break down with age, leading to an altered epigenome and increased eAge. The NSD1 mutation Martin‐Herranz et al. observed might simulate an ageing affect that occurs naturally. An updated study with more samples (particularly of Sotos syndrome) is required to corroborate their findings (Martin‐Herranz et al., 2019).

## 6 NON‐HUMAN EPIGENETIC AGE PREDICTORS

Since the advent of DNAm age prediction for humans, age predictors have been created for other species; mice (Table 4), rats (Horvath, Singh, et al., 2020; Levine et al., 2020), dogs (Thompson et al., 2017; Wang et al., 2020), wolves (Thompson et al., 2017), humpback whales (Polanowski et al., 2014), chimpanzees (Guevara et al., 2020; Ito et al., 2018), marmosets (Horvath, Zoller, Haghani, Lu, et al., 2020), naked mole rats (Lowe et al., 2020), sea bass (Anastasiadi & Piferrer, 2020) and zebrafish (Mayne et al., 2020) (see Table 5 for a list of non‐human/mouse epigenetic clocks). In 2017, three mouse epigenetic clocks were developed primarily using reduced representation bisulphite sequencing (RRBS) data. Wang et al. 2017 used 148 CpGs from liver tissue (using both RRBS and whole genome bisulphite data, WGBS) and found a moderate conservation of age‐related CpGs between human and mouse. Their clock also showed an age reduction for calorie restriction, rapamycin and Prop1^df/df^ dwarfism (which results in lifespan extension up to 1.5 fold) (Brown‐Borg et al., 1996; Cole et al., 2017; Wang et al., 2017). Petkovich et al. built a mouse epigenetic clock using 90 CpGs from blood and detected that calorie restriction reduces epigenetic age according to their clock (Petkovich et al., 2017). The first mouse multi‐tissue age predictor was constructed based on 329 unique CpGs with a median absolute error of 3.33 weeks, mainly trained on young‐ and middle‐aged mice (0.2–9.5 months) (Stubbs et al., 2017). A recent multi‐tissue age predictor in mouse has been developed by Meer et al. that uses 435 CpGs, and predicts age across a wide age range (1–35 months) (Meer et al., 2018). It operates on multiple tissues including blood, liver, brain and heart (Meer et al., 2018). Thompson et al. created four mouse RRBS clocks to compare statistical methods and found the most accurate clock resulting from elastic net regression (Thompson et al., 2018).

**TABLE 4:** Mouse epigenetic clocks

Clock	Number of CpGs	Correlation (*R* ^2^)	Generation of error estimate (Type of validation data set)	Number of samples in training data	Method used to find age‐associated CpGs	Age range of training samples (Months)	Cell types/Tissue used for training	Reference
Wang	107	0.91	Independent validation data set	148	Elastic net	0.2–26	Liver	Wang et al. (2017)
Petkovich	90	>0.90	Independent validation data set	141	Elastic net	3–35	Partial blood	Petkovich et al. (2017)
Stubbs Multi‐Tissue	329	0.7	Training data sets partitioned and mixed with two external data sets to make up validation data set	129	Elastic net	0.2–9.5	Liver, lung, heart, muscle, spleen, cerebellum, cortex	Stubbs et al. (2017)
Meer	435	0.89	Random segregation of validation data set from training	~333	Elastic net	0.2–35	Blood, heart, cortex, liver, lung, muscle, spleen, cerebellum, pro B cells, follicular B cells	Meer et al. (2018)
Thompson All CpGs (Ridge)	582	0.79	Leave‐one‐batch‐out	893	Ridge Regression	0–30	Various tissues including adipose, blood, kidney, liver, lung, muscle, spleen	Thompson et al. (2018)
Thompson All CpGs (Elastic Net)	582	0.82	Leave‐one‐batch‐out	893	Elastic net	0–30	""	Thompson et al. (2018)
Thompson Conserved CpGs (Ridge)	273	0.64	Leave‐one‐batch‐out	893	Ridge Regression	0–30	""	Thompson et al. (2018)
Thompson Conserved CpGs (Elastic Net)	273	0.68	Leave‐one‐batch‐out	893	Elastic net	0–30	""	Thompson et al. (2018)
Wood Mouse Clock	9	0.88	Same data set used for training	48	LASSO	3–16	Ear punch samples	Little et al. (2020)

Note

All clocks were trained on mouse RRBS data (with the exception of Wang et al., which used both RRBS and WGBS, and the Wood Mouse Clock, which used a targeted PCR approach combined with Oxford Nanopore).

**TABLE 5:** Studies that have developed epigenetic clocks for non‐human and non‐mouse species (with the exception of dual species clocks)

Study	Species	Platform
Polanowski et al. (2014)	Humpback whale	Bisulphite pyrosequencing
Thompson et al. (2017)	Dogs, wolves	RRBS
Wang et al. (2020)	Mouse, dogs	Syntenic Bisulfite Sequencing
Ito et al. (2018)	Chimpanzee	Bisulphite pyrosequencing
Guevara et al. (2020)	Chimpanzee, human	Human Illumina 850K array
Lowe et al. (2020)	Naked mole rat	Bisulphite PCR
Anastasiadi and Piferrer (2020)	Seabass	Multiplex bisulphite sequencing
Mayne et al. (2020)	Zebrafish	RRBS
Levine et al. (2020)	Rat	RRBS
Horvath, Singh, et al. (2020)	Rat, human	HorvathMammalMethylChip40
Horvath, Zoller, Haghani, Lu, et al. (2020)	Marmoset	HorvathMammalMethylChip40
Horvath, Zoller, Haghani, Janinska, et al. (2020)	Macaque, human	HorvathMammalMethylChip40
Horvath, Haghani, et al. (2020)	Baboon, marmoset, vervet monkey, macaque, human	HorvathMammalMethylChip40
Jasinska et al. (2020)	Vervet monkey, human	HorvathMammalMethylChip40
Wilkinson et al. (2021)	Bat	HorvathMammalMethylChip40
Raj et al. (2020)	Cat, human	HorvathMammalMethylChip40
Prado et al. (2020)	Elephant, human	HorvathMammalMethylChip40
Bors et al. (2021)	Beluga whale	HorvathMammalMethylChip40
Pinho et al. (2021)	Marmot	HorvathMammalMethylChip40
Sailer et al. (2020)	Prairie vole, human	HorvathMammalMethylChip40
Sugrue et al. (2021)	Sheep, human	HorvathMammalMethylChip40
Kordowitzki et al. (2021)	Cattle, human	HorvathMammalMethylChip40
Lemaître et al. (2020)	Deer	HorvathMammalMethylChip40
Schachtschneider et al. (2020)	Pig, human	HorvathMammalMethylChip40

The Wang, Stubbs and Petkovich mouse clocks mentioned here show little overlap in CpGs used (Field et al., 2018). This is probably due to the variability of RRBS data, where the regional genome coverage differs between protocols and enzymes used, rather than different statistical methods applied (Field et al., 2018; Thompson et al., 2018). Transferability of these clocks to data sets outside of the original studies has therefore been difficult. WGBS at a high enough coverage for eAge prediction is expensive, and most mouse clocks are trained on RRBS. Another alternative has been developed by FOXO BioScience, who have collaborated with Van Andel Institute and Illumina to create a cost‐effective Infinium Mouse Methylation Array (FOXO BioScience, 2020).

Indeed, other studies have created a similar custom array to accurately predict age in model organisms. Currently available as a preprint, the Horvath laboratory has published an epigenetic clock that works on both rats and humans (Horvath, Singh, et al., 2020). This was created using a custom Illumina methylation array called the HorvathMammalMethylChip40, made up of 36,000 CpGs conserved among 50 mammalian species (Arneson et al., 2021; Horvath, Singh, et al., 2020). The MAE for human and rat data was 0.03, and a correlation of 0.95. Three single tissue clocks were also created for rat liver, brain and blood, as well as a multi‐tissue clock combining all three tissues (Horvath, Singh, et al., 2020). Another preprint has been released of a sheep epigenetic clock, using the same array, with a median error of 5.1 months (~3.5–4.2% of expected sheep lifespan). The study reported that castrated sheep had a higher age acceleration than age‐matched controls, and a dual human and sheep clock was constructed with an additional 1,848 human samples (Sugrue et al., 2020). A rat clock has also been developed using 134 RRBS whole blood samples (Levine et al., 2020). Elastic net selected 68 CpGs, and had a correlation of *r* = 0.9 in their test data set. It appears to work in mice, where it predicted reduced age acceleration after calorie restriction.

Many age‐associated CpGs are conserved between different species (Horvath, 2013; Horvath, Singh, et al., 2020; Wang et al., 2017, 2020), meaning the development of pan‐species clocks is plausible. For example, an epigenetic clock has been created using 394 CpGs from modules of developmental genes with conserved, age‐related methylation changes, between mouse, human and dogs (Wang et al., 2020). Recent preprints have shown various universal pan‐tissue epigenetic clocks that predict age across 9 tissue types from 128 different mammalian species (Lu et al., 2021), and models that predict maximum lifespan, gestation time and sexual maturity (Li et al., 2021). The CpGs used to construct the clocks were also associated with genes that are enriched during mammalian development (Lu et al., 2021). These clocks further enforce the idea that ageing is a conserved evolutionary process intertwined with mammalian development.

## 7 CONCLUSION

eAge prediction is a powerful approach that has revolutionised experimental gerontology. As the number and diversity of epigenetic clocks increases, so too does our understanding of biological age. Depending on how these clocks are constructed, they appear to capture different aspects of ageing. These differences depend on the tissues, number of samples, age range and algorithms used in their construction.

Whether the change of methylation is causal to ageing remains to be shown and herein lies a caveat studying diseases or interventions that directly affect DNAm. Studying a process that interacts with DNAm might alter age prediction, without changing the actual ageing trajectory. For example, it is possible that a global increase or decrease in methylation caused either by technical errors (Olova et al., 2018) or mutations in oncogenes (such as *DNMT3A* or *TET2*; Robertson et al., 2019), could result in false‐positive shifts of eAge. It remains to be tested how stable epigenetic clocks are against global sweeps of DNAm.

Linear models have proven effective predicting eAge of individuals between the ages of 20 and ~70, but drop in accuracy outside of these ages. Clocks trained on specific age groups, such as PedBE, are valid approaches to this issue (McEwen et al., 2020). Alternative non‐linear models may be better aligned with the actual trajectories of methylation changes with age. However, it is also possible that training eAge on chAge alone is not enough to explain biological age, as demonstrated by composite approaches such as PhenoAge and GrimAge clocks.

Multiple studies have shown that it is possible to build accurate minimised clocks using only a few highly age‐associated CpGs (Cho et al., 2017; Daunay et al., 2019; Dias, Cordeiro, Pereira, et al., 2020; Jung et al., 2019; Weidner et al., 2014; Zbiec´‐Piekarska et al., 2015). Many of these clocks use CpGs nearby *ELOVL2* and *FHL2*, and work in saliva and blood (Cho et al., 2017; Dias, Cordeiro, Pereira, et al., 2020; Jung et al., 2019; Zbiec´‐Piekarska et al., 2015). They have yet to be tested in other scenarios, such as clinical applications. On the other hand, clocks might be more robust when utilising a large number of CpGs (Boroni et al., 2020; Zhang, Kamath ,et al., 2019).

The approach used by Horvath to develop epigenetic clocks has spawned not only an abundance of similar DNAm age predictors, but also other novel approaches, such as transcriptional (Bryois et al., 2017; Peters et al., 2015), proteomic (Lehallier et al., 2019; Tanaka et al., 2018) and cellular biophysical/biomolecular (Phillip et al., 2017) clocks. Indeed, DNAm can be regressed with health co‐factors such as smoking and alcohol consumption to produce predictors of complex traits and mortality (McCartney et al., 2018). While DNAm is one of the most accurate and versatile biomarkers for ageing and disease, our understanding of it is still developing. Perhaps looking at DNAm in combination with other non‐DNAm based biomarkers will broaden our understanding and predictive power of biological ageing and mortality. Composite clocks such as PhenoAge and GrimAge are first steps in that direction. Transcription clocks may reveal regulators of biological ageing, for example, if key ageing genes are found to be linked with eAge either by correlating with age acceleration or directly with methylation changes of key clock CpGs.

To us, the key areas to emerge will be in a) understanding the different aspects of ageing captured by distinct clocks and b) testing causality of DNAm in age acceleration through interventional epigenetics and other approaches. As epigenetic clocks become more sophisticated and commonplace, caution must be considered when inferring the biological significance of age acceleration. Research must continue regarding the nature of eAge and the aspects of ageing captured.

## CONFLICT OF INTEREST

None declared.

## AUTHOR CONTRIBUTIONS

D.J.S. and T.C. both wrote and conceived the manuscript and are both corresponding authors.

## References

[acel13452-bib-0001] Al Muftah, W. A., Al‐Shafai, M., Zaghlool, S. B., Visconti, A., Tsai, P.‐C., Kumar, P., Spector, T., Bell, J., Falchi, M., & Suhre, K. (2016). Epigenetic associations of type 2 diabetes and BMI in an Arab population. Clinical Epigenetics, 8(1). 10.1186/s13148-016-0177-6 PMC4730771 26823690

[acel13452-bib-0002] Aliferi, A., Ballard, D., Gallidabino, M. D., Thurtle, H., Barron, L., & Syndercombe Court, D. (2018). DNA methylation‐based age prediction using massively parallel sequencing data and multiple machine learning models. Forensic Science International: Genetics, 37, 215–226. 10.1016/j.fsigen.2018.09.003 30243148

[acel13452-bib-0003] Anastasiadi, D., & Piferrer, F. (2020). A clockwork fish: Age prediction using DNA methylation‐based biomarkers in the European seabass. Molecular Ecology Resources, 20(2), 387–397. 10.1111/1755-0998.13111 31674713

[acel13452-bib-0004] Angermueller, C., Clark, S. J., Lee, H. J., Macaulay, I. C., Teng, M. J., Hu, T. X., Krueger, F., Smallwood, S. A., Ponting, C. P., Voet, T., Kelsey, G., Stegle, O., & Reik, W. (2016). Parallel single‐cell sequencing links transcriptional and epigenetic heterogeneity. Nature Methods, 13(3), 229–232. 10.1038/nmeth.3728 26752769 PMC4770512

[acel13452-bib-0005] Arneson, A., Haghani, A., Thompson, M. J., Pellegrini, M., Kwon, S. B., Vu, H., Yao, M., Li, C. Z., Lu, A. T., Barnes, B., Hansen, K. D., Zhou, W., Breeze, C. E., Ernst, J., & Horvath, S. (2021). A mammalian methylation array for profiling methylation levels at conserved sequences. bioRxiv, 2021.01.07.425637. 10.1038/s41467-022-28355-z PMC8831611 35145108

[acel13452-bib-0006] Bacalini, M. G., Deelen, J., Pirazzini, C., De Cecco, M., Giuliani, C., Lanzarini, C., Ra‐vaioli, F., Marasco, E., Van Heemst, D., Suchiman, H. E. D., Slieker, R., Giampieri, E., Recchioni, R., Marcheselli, F., Salvioli, S., Vitale, G., Olivieri, F., Spijkerman, A. M., DollCrossed, M. E., … Garagnani, P. (2017). Systemic age‐associated DNA hypermethylation of ELOVL2 Gene. In vivo and in vitro evidences of a cell replication process. Journals of Gerontology ‐ Series A Biological Sciences and Medical Sciences, 72(8), 1015–1023. 10.1093/gerona/glw185 PMC5861890 27672102

[acel13452-bib-0007] Baker, G. T., & Sprott, R. L. (1988). Biomarkers of aging. Experimental Gerontology, 23(4‐5), 223–239. 10.1016/0531-5565(88)90025-3 3058488

[acel13452-bib-0008] Bell, C. G., Lowe, R., Adams, P. D., Baccarelli, A. A., Beck, S., Bell, J. T., Christensen, B. C., Gladyshev, V. N., Heijmans, B. T., Horvath, S., Ideker, T., Issa, J.‐P., Kelsey, K. T., Marioni, R. E., Reik, W., Relton, C. L., Schalkwyk, L. C., Teschendorff, A. E., Wagner, W., … Rakyan, V. K. (2019). DNA methylation aging clocks: Challenges and recommendations. Genome Biology, 20(1), 1–24. 10.1186/s13059-019-1824-y 31767039 PMC6876109

[acel13452-bib-0200] Belsky, D. W., Caspi, A., Arseneault, L., Baccarelli, A., Corcoran, D., Gao, X., Hannon, E., Harrington, H. L., Rasmussen, L. J., Houts, R., Huffman, K., Kraus, W. E., Kwon, D., Mill, J., Pieper, C. F., Prinz, J., Poulton, R., Schwartz, J., Sugden, K., … Moffitt, T. E. (2020). Quantification of the pace of biological aging in humans through a blood test, the DunedinPoAm DNA methylation algorithm. eLife, 9, 1–56. 10.7554/eLife.54870 PMC7282814 32367804

[acel13452-bib-0010] Belsky, D. W., Caspi, A., Houts, R., Cohen, H. J., Corcoran, D. L., Danese, A., Harrington, H., Israel, S., Levine, M. E., Schaefer, J. D., Sugden, K., Williams, B., Yashin, A. I., Poulton, R., & Moffitt, T. E. (2015). Quantification of biological aging in young adults. Proceedings of the National Academy of Sciences of the United States of America, 112(30), E4104–E4110. 26150497 10.1073/pnas.1506264112 PMC4522793

[acel13452-bib-0011] Benayoun, B. A., Pollina, E. A., & Brunet, A. (2015). Epigenetic regulation of ageing: Linking environmental inputs to genomic stability. Nature Reviews Molecular Cell Biology, 16(10), 593–610. 10.1038/nrm4048 26373265 PMC4736728

[acel13452-bib-0012] Bergsma, T., & Rogaeva, E. (2020). DNA methylation clocks and their predictive capacity for aging phenotypes and healthspan. Neuroscience Insights, 15, 263310552094222. 10.1177/2633105520942221 PMC7376380 32743556

[acel13452-bib-0013] Binder, A. M., Corvalan, C., Mericq, V., Pereira, A., Santos, J. L., Horvath, S., Shepherd, J., & Michels, K. B. (2018). Faster ticking rate of the epigenetic clock is associated with faster pubertal development in girls. Epigenetics, 13(1), 85–94. 10.1080/15592294.2017.1414127 29235933 PMC5836971

[acel13452-bib-0014] Blüher, M., Kahn, B. B., Kahn, C. R. (2003). Extended longevity in mice lacking the insulin receptor in adipose tissue. Science, 299(5606), 572–574. 12543978 10.1126/science.1078223

[acel13452-bib-0015] Bocklandt, S., Lin, W., Sehl, M. E., Sánchez, F. J., Sinsheimer, J. S., Horvath, S., & Vilain, E. (2011). Epigenetic predictor of age. PLoS One, 6(6), e14821. 10.1371/journal.pone.0014821 21731603 PMC3120753

[acel13452-bib-0016] Boroni, M., Zonari, A., Reis de Oliveira, C., Alkatib, K., Ochoa Cruz, E. A., Brace, L. E., & Lott de Carvalho, J. (2020). Highly accurate skin‐specific methylome analysis algorithm as a platform to screen and validate therapeutics for healthy aging. Clinical Epigenetics, 12, 1. 10.1186/s13148-020-00899-1 PMC7359467 32660606

[acel13452-bib-0017] Bors, E. K., Baker, C. S., Wade, P. R., O'Neill, K. B., Shelden, K. E. W., Thompson, M. J., Fei, Z., Jarman, S., & Horvath, S. (2021). An epigenetic clock to estimate the age of living beluga whales. Evolutionary Applications, 14(5), 1263–1273. 10.1111/eva.13195 34025766 PMC8127720

[acel13452-bib-0018] Breitling, L. P., Saum, K.‐U., Perna, L., Schöttker, B., Holleczek, B., & Brenner, H. (2016). Frailty is associated with the epigenetic clock but not with telomere length in a German cohort. Clinical Epigenetics, 8(1), 1–8. 10.1186/s13148-016-0186-5 26925173 PMC4768341

[acel13452-bib-0019] Brown‐Borg, H. M., Borg, K. E., Meliska, C. J., & Bartke, A. (1996). Dwarf mice and the ageing process. Nature, 384(6604), 33. 10.1038/384033a0 8900272

[acel13452-bib-0020] Bryois, J., Buil, A., Ferreira, P. G., Panousis, N. I., Brown, A. A., Viñuela, A., Planchon, A., Bielser, D., Small, K., Spector, T., & Dermitzakis, E. T. (2017). Time‐dependent genetic effects on gene expression implicate aging processes. Genome Research, 27(4), 545–552. 10.1101/gr.207688.116 28302734 PMC5378173

[acel13452-bib-0021] Chambers, J. C., Loh, M., Lehne, B., Drong, A., Kriebel, J., Motta, V., Wahl, S., Elliott, H. R., Rota, F., Scott, W. R., Zhang, W., Tan, S.‐T., Campanella, G., Chadeau‐Hyam, M., Yengo, L., Richmond, R. C., Adamowicz‐Brice, M., Afzal, U., Bozaoglu, K., … Kooner, J. S. (2015). Epigenome‐wide association of DNA methylation markers in peripheral blood from Indian Asians and Europeans with incident type 2 diabetes: A nested case‐control study. The Lancet Diabetes and Endocrinology, 3(7), 526–534. 10.1016/S2213-8587(15)00127-8 26095709 PMC4724884

[acel13452-bib-0022] Chandra, T., & Kirschner, K. (2016). Chromosome organisation during ageing and senescence. Current Opinion in Cell Biology, 40, 161–167. 10.1016/j.ceb.2016.03.020 27101466

[acel13452-bib-0023] Chen, B. H., Marioni, R. E., Colicino, E., Peters, M. J., Ward‐Caviness, C. K., Tsai, P.‐C., Roetker, N. S., Just, A. C., Demerath, E. W., Guan, W., Bressler, J., Fornage, M., Studenski, S., Vandiver, A. R., Moore, A. Z., Tanaka, T., Kiel, D. P., Liang, L., Vokonas, P., … Horvath, S. (2016). DNA methylation‐based measures of biological age: Meta‐analysis predicting time to death. Aging, 8(9), 1844–1865. 10.18632/aging.101020 27690265 PMC5076441

[acel13452-bib-0024] Chen, D., Chao, D. L., Rocha, L., Kolar, M., Nguyen Huu, V. A., Krawczyk, M., Dasyani, M., Wang, T., Jafari, M., Jabari, M., Ross, K. D., Saghatelian, A., Hamilton, B. A., Zhang, K., & Skowronska‐Krawczyk, D. (2020). The lipid elongation enzyme ELOVL2 is a molecular regulator of aging in the retina. Aging Cell, 19, 2. 10.1111/acel.13100 PMC6996962 31943697

[acel13452-bib-0025] Chen, L. I., Dong, Y., Bhagatwala, J., Raed, A., Huang, Y., & Zhu, H. (2019). Effects of Vitamin D 3 supplementation on epigenetic aging in overweight and obese African Americans with suboptimal Vitamin D status: A randomized clinical trial. Journals of Gerontology ‐ Series A Biological Sciences and Medical Sciences, 74(1), 91–98. 10.1093/gerona/gly223 PMC6612014 30256915

[acel13452-bib-0026] Cho, S., Jung, S.‐E., Hong, S. R., Lee, E. H., Lee, J. H., Lee, S. D., & Lee, H. Y. (2017). Independent validation of DNA‐based approaches for age prediction in blood. Forensic Science International: Genetics, 29, 250–256. 10.1016/j.fsigen.2017.04.020 28511095

[acel13452-bib-0027] Choufani, S., Cytrynbaum, C., Chung, B. H. Y., Turinsky, A. L., Grafodatskaya, D., Chen, Y. A., Cohen, A. S. A., Dupuis, L., Butcher, D. T., Siu, M. T., Luk, H. M., Lo, I. F. M., Lam, S. T. S., Caluseriu, O., Stavropoulos, D. J., Reardon, W., Mendoza‐Londono, R., Brudno, M., Gibson, W. T., … Weksberg, R. (2015). NSD1 mutations generate a genome‐wide DNA methylation signature. Nature Communications, 6(1). 10.1038/ncomms10207 PMC4703864 26690673

[acel13452-bib-0028] Christiansen, L., Lenart, A., Tan, Q., Vaupel, J. W., Aviv, A., McGue, M., & Christensen, K. (2016). DNA methylation age is associated with mortality in a longitudinal Danish twin study. Aging Cell, 15(1), 149–154. 10.1111/acel.12421 26594032 PMC4717264

[acel13452-bib-0029] Cole, J. J., Robertson, N. A., Rather, M. I., Thomson, J. P., McBryan, T., Sproul, D., Wang, T., Brock, C., Clark, W., Ideker, T., Meehan, R. R., Miller, R. A., Brown‐Borg, H. M., & Adams, P. D. (2017). Diverse interventions that extend mouse lifespan suppress shared age‐associated epigenetic changes at critical gene regulatory regions. Genome Biology, 18(1). 10.1186/s13059-017-1185-3 PMC5370462 28351383

[acel13452-bib-0034] Correia Dias, H., Cordeiro, C., Corte Real, F., Cunha, E., & Manco, L. (2020). Age Estimation Based on DNA Methylation Using Blood Samples From Deceased Individuals. Journal of Forensic Sciences, 65(2), 465–470. 10.1111/1556-4029.14185 31490551

[acel13452-bib-0031] Cypris, O., Eipel, M., Franzen, J., Rösseler, C., Tharmapalan, V., Kuo, C.‐C., Vieri, M., Nikolić, M., Kirschner, M., Brümmendorf, T. H., Zenke, M., Lampert, A., Beier, F., & Wagner, W. (2020). PRDM8 reveals aberrant DNA methylation in aging syndromes and is relevant for hematopoietic and neuronal differentiation. Clinical Epigenetics, 12(1), 125. 10.1186/s13148-020-00914-5 32819411 PMC7439574

[acel13452-bib-0032] Daunay, A., Baudrin, L. G., Deleuze, J.‐F., & How‐Kit, A. (2019). Evaluation of six blood‐based age prediction models using DNA methylation analysis by pyrosequencing. Scientific Reports, 9, 1. 10.1038/s41598-019-45197-w 31222117 PMC6586942

[acel13452-bib-0033] Declerck, K., & Berghe, W. V. (2018). Back to the future: Epigenetic clock plasticity towards healthy aging. Mechanisms of Ageing and Development, 174, 18–29. 10.1016/j.mad.2018.01.002 29337038

[acel13452-bib-0335] Correia Dias, H., Cordeiro, C., Corte Real, F., Cunha, E., Manco, L. (2020). Age Estimation Based on DNA Methylation Using Blood Samples From Deceased Individuals. Journal of Forensic Sciences, 65(2), 465–470. 10.1111/1556-4029.14185 31490551

[acel13452-bib-0035] Dias, H. C., Cordeiro, C., Pereira, J., Pinto, C., Real, F. C., Cunha, E., & Manco, L. (2020). DNA methylation age estimation in blood samples of living and deceased individuals using a multiplex SNaPshot assay. Forensic Science International, 311, 110267. 10.1016/j.forsciint.2020.110267 32325350

[acel13452-bib-0030] Dias, H. C., Cunha, E., Corte Real, F., & Manco, L. (2020). Age prediction in living: Forensic epigenetic age estimation based on blood samples. Legal Medicine, 47, 101763. 10.1016/j.legalmed.2020.101763 32721866

[acel13452-bib-0036] Eipel, M., Mayer, F., Arent, T., Ferreira, M. R. P., Birkhofer, C., Gerstenmaier, U., Costa, I. G., Ritz‐Timme, S., & Wagner, W. (2016). Epigenetic age predictions based on buccal swabs are more precise in combination with cell type‐specific DNA methylation signatures. Aging, 8(5), 1034–1048. 10.18632/aging.100972 27249102 PMC4931852

[acel13452-bib-0037] El Khoury, L. Y., Gorrie‐Stone, T., Smart, M., Hughes, A., Bao, Y., Andrayas, A., Burrage, J., Hannon, E., Kumari, M., Mill, J., & Schalkwyk, L. C. (2019). Systematic underestimation of the epigenetic clock and age acceleration in older subjects. Genome Biology, 20, 1. 10.1186/s13059-019-1810-4 31847916 PMC6915902

[acel13452-bib-0038] Field, A. E., Robertson, N. A., Wang, T., Havas, A., Ideker, T., & Adams, P. D. (2018). Molecular cell review DNA methylation clocks in aging: Categories, causes and consequences. Molecular Cell, 71(6), 882–895. 30241605 10.1016/j.molcel.2018.08.008 PMC6520108

[acel13452-bib-0039] Fleckhaus, J., & Schneider, P. M. (2020). Novel multiplex strategy for DNA methylation‐based age prediction from small amounts of DNA via Pyrosequencing. Forensic Science International: Genetics, 44, 102189. 31648151 10.1016/j.fsigen.2019.102189

[acel13452-bib-0040] FOXO BioScience (2020). FOXO BioScience, Formerly Known as Life Epigenetics, An nounces New Infinium Mouse Methylation Array in Strategic Collaboration with Van Andel Institute. https://www.prnewswire.com/news‐releases/foxo‐bioscience‐formerly‐known‐as‐life‐epigenetics‐announces‐new‐infinium‐mouse‐methylation‐array‐in‐strategic‐collaboration‐with‐van‐andel‐institute‐301038822.html

[acel13452-bib-0041] Frenck, R. W., Blackburn, E. H., & Shannon, K. M. (1998). The rate of telomere sequence loss in human leukocytes varies with age. Proceedings of the National Academy of Sciences of the United States of America, 95, 5607–5610. 9576930 10.1073/pnas.95.10.5607 PMC20425

[acel13452-bib-0042] Galkin, F., Mamoshina, P., Aliper, A., Putin, E., Moskalev, V., Gladyshev, V. N., & Zhavoronkov, A. (2020). Human gut microbiome aging clock based on taxonomic profiling and deep learning. iScience, 23(6), 101199. 32534441 10.1016/j.isci.2020.101199 PMC7298543

[acel13452-bib-0043] Galkin, F., Mamoshina, P., Kochetov, K., Sidorenko, D., & Zhavoronkov, A. (2021). DeepMAge: A methylation aging clock developed with deep learning. Aging and Disease, 12(5), 1252. 34341706 10.14336/AD.2020.1202 PMC8279523

[acel13452-bib-0044] Gao, X. U., Jia, M., Zhang, Y., Breitling, L. P., & Brenner, H. (2015). DNA methylation changes of whole blood cells in response to active smoking exposure in adults: A systematic review of DNA methylation studies. Clinical Epigenetics, 7(1), 113. 10.1186/s13148-015-0148-3 26478754 PMC4609112

[acel13452-bib-0045] Garagnani, P., Bacalini, M. G., Pirazzini, C., Gori, D., Giuliani, C., Mari, D., Di Blasio, A. M., Gentilini, D., Vitale, G., Collino, S., Rezzi, S., Castellani, G., Capri, M., Salvioli, S., & Franceschi, C. (2012). Methylation ofELOVL2gene as a new epigenetic marker of age. Aging Cell, 11(6), 1132–1134. 10.1111/acel.12005 23061750

[acel13452-bib-0046] Grant, C. D., Jafari, N., Hou, L., Li, Y., Stewart, J. D., Zhang, G., Lamichhane, A., Manson, J. A. E., Baccarelli, A. A., Whitsel, E. A., & Conneely, K. N. (2017). A longitudinal study of DNA methylation as a potential mediator of age‐related diabetes risk. GeroScience, 39(5‐6), 475. 10.1007/s11357-017-0001-z 29159506 PMC5745220

[acel13452-bib-0047] Guevara, E. E., Lawler, R. R., Staes, N., White, C. M., Sherwood, C. C., Ely, J. J., Hopkins, W. D., & Bradley, B. J. (2020). Age‐associated epigenetic change in chimpanzees and humans. Philosophical Transactions of the Royal Society B: Biological Sciences, 375(1811), 20190616. 10.1098/rstb.2019.0616 PMC7540949 32951551

[acel13452-bib-0048] Hannum, G., Guinney, J., Zhao, L., Zhang, L. I., Hughes, G., Sadda, S. V., Klotzle, B., Bibikova, M., Fan, J.‐B., Gao, Y., Deconde, R., Chen, M., Rajapakse, I., Friend, S., Ideker, T., & Zhang, K. (2013). Genome‐wide methylation profiles reveal quantitative views of human aging rates. Molecular Cell, 49(2), 359–367. 10.1016/j.molcel.2012.10.016 23177740 PMC3780611

[acel13452-bib-0049] Harley, C. B., Bruce Futcher, A., & Greider, C. W. (1990). Telomeres shorten during ageing of human fibroblasts. Nature, 345(6274), 458–460. 10.1038/345458a0 2342578

[acel13452-bib-0050] Hastie, N. D., Dempster, M., Dunlop, M. G., Thompson, A. M., Green, D. K., & Allshire, R. C. (1990). Telomere reduction in human colorectal carcinoma and with ageing. Nature, 346(6287), 866–868. 10.1038/346866a0 2392154

[acel13452-bib-0051] Hayflick, L. (1965). The limited in vitro lifetime of human diploid cell strains. Experimental Cell Research, 37(3), 614–636. 10.1016/0014-4827(65)90211-9 14315085

[acel13452-bib-0052] Hayflick, L., & Moorhead, P. S. (1961). The serial cultivation of human diploid cell strains. Experimental Cell Research, 25(3), 585–621. 10.1016/0014-4827(61)90192-6 13905658

[acel13452-bib-0053] Hendrich, B., Hardeland, U., Ng, H.‐H., Jiricny, J., & Bird, A. (1999). The thymine glycosylase MBD4 can bind to the product of deamination at methylated CpG sites. Nature, 401(6750), 301–304. 10.1038/45843 10499592

[acel13452-bib-0054] Hernando‐Herraez, I., Evano, B., Stubbs, T., Commere, P.‐H., Jan Bonder, M., Clark, S., Andrews, S., Tajbakhsh, S., & Reik, W. (2019). Ageing affects DNA methylation drift and transcriptional cell‐to‐cell variability in mouse muscle stem cells. Nature Communications, 10(1), 4361. 10.1038/s41467-019-12293-4 PMC6761124 31554804

[acel13452-bib-0055] Higgins‐Chen, A. T., Boks, M. P., Vinkers, C. H., Kahn, R. S., & Levine, M. E. (2020). Schizophrenia and epigenetic aging biomarkers: Increased mortality, reduced cancer risk, and unique clozapine effects. Biological Psychiatry, 88(3), 224–235. 10.1016/j.biopsych.2020.01.025 32199607 PMC7368835

[acel13452-bib-0056] Hillary, R. F., Stevenson, A. J., McCartney, D. L., Campbell, A., Walker, R. M., Howard, D. M., Ritchie, C. W., Horvath, S., Hayward, C., McIntosh, A. M., Porteous, A. M., Deary, A. M., Evans, K. L., & Marioni, R. E. (2020). Epigenetic measures of ageing predict the prevalence and incidence of leading causes of death and disease burden. Clinical Epigenetics, 12(1), 115. 32736664 10.1186/s13148-020-00905-6 PMC7394682

[acel13452-bib-0057] Hiyama, E., & Hiyama, K. (2007). Telomere and telomerase in stem cells. British Journal of Cancer, 96(7), 1020–1024. 17353922 10.1038/sj.bjc.6603671 PMC2360127

[acel13452-bib-0058] Holzenberger, M., Dupont, J., Ducos, B., Leneuve, P., Géloën, A., Even, P. C., Cervera, P., & Le Bouc, Y. (2003). IGF‐1 receptor regulates lifespan and resistance to oxidative stress in mice. Nature, 421(6919), 182–187. 12483226 10.1038/nature01298

[acel13452-bib-0059] Horvath, S. (2013). DNA methylation age of human tissues and cell types. Genome Biology, 14. 10.1186/gb-2013-14-10-r115 PMC4015143 24138928

[acel13452-bib-0060] Horvath, S. (2015). Erratum to DNA methylation age of human tissues and cell types [Genome Biology, 14, R115, (2013)]. Genome Biology, 16(1), 1–5. 25968125 10.1186/s13059-015-0649-6 PMC4427927

[acel13452-bib-0065] Horvath, S., Erhart, W., Brosch, M., Ammerpohl, O., von Schonfels, W., Ahrens, M., Heits, N., Bell, J. T., Tsai, P.‐C., Spector, T. D., Deloukas, P., Siebert, R., Sipos, B., Becker, T., Rocken, C., Schafmayer, C., & Hampe, J. (2014). Obesity accelerates epigenetic aging of human liver. Proceedings of the National Academy of Sciences of the United States of America, 111(43), 15538–15543. 10.1073/pnas.1412759111 25313081 PMC4217403

[acel13452-bib-0066] Horvath, S., Garagnani, P., Bacalini, M. G., Pirazzini, C., Salvioli, S., Gentilini, D., Di Blasio, A. M., Giuliani, C., Tung, S., Vinters, H. V., & Franceschi, C. (2015a). Accelerated epigenetic aging in Down syndrome. Aging Cell, 14(3), 491–495. 10.1111/acel.12325 25678027 PMC4406678

[acel13452-bib-0067] Horvath, S., Gurven, M., Levine, M. E., Trumble, B. C., Kaplan, H., Allayee, H., Ritz, B. R., Chen, B., Lu, A. T., Rickabaugh, T. M., Jamieson, B. D., Sun, D., Li, S., Chen, W., Quintana‐Murci, L., Fagny, M., Kobor, M. S., Tsao, P. S., Reiner, A. P., … Assimes, T. L. (2016a). An epigenetic clock analysis of race/ethnicity, sex, and coronary heart disease. Genome Biology, 17(1), 171. 10.1186/s13059-016-1030-0 27511193 PMC4980791

[acel13452-bib-0062] Horvath, S., Haghani, A., Zoller, J. A., Ernst, J., Pellegrini, M., Jasinska, A. J., Mattison, J. A., Salmon, A. B., Raj, K., Jenkins, S., Li, C., & Nathanielsz, P. W. (2020). DNA methylation study of age and sex in baboons and four other primates. bioRxiv, 2020.11.29.402891.

[acel13452-bib-0068] Horvath, S., Langfelder, P., Kwak, S., Aaronson, J., Rosinski, J., Vogt, T. F., Eszes, M., Faull, R. L. M., Curtis, M. A., Waldvogel, H. J., Choi, O.‐W., Tung, S., Vinters, H. V., Coppola, G., & Yang, X. W. (2016b). Huntington’s disease accelerates epigenetic aging of human brain and disrupts DNA methylation levels. Aging, 8(7), 1485–1512. 10.18632/aging.101005 27479945 PMC4993344

[acel13452-bib-0069] Horvath, S., & Levine, A. J. (2015). HIV‐1 infection accelerates age according to the epigenetic clock. Journal of Infectious Diseases, 212(10), 1563–1573. 10.1093/infdis/jiv277 PMC4621253 25969563

[acel13452-bib-0070] Horvath, S., Lu, A. T., Cohen, H., & Raj, K. (2019). Rapamycin retards epigenetic ageing of keratinocytes independently of its effects on replicative senescence, proliferation and differentiation. Aging, 11(10), 3238–3249. 10.18632/aging.101976 31136303 PMC6555449

[acel13452-bib-0071] Horvath, S., Mah, V., Lu, A. T., Woo, J. S., Choi, O.‐W., Jasinska, A. J., Riancho, J. A., Tung, S., Coles, N. S., Braun, J., Vinters, H. V., & Coles, L. S. (2015c). The cerebellum ages slowly according to the epigenetic clock. Aging, 7(5), 294–306. 10.18632/aging.100742 26000617 PMC4468311

[acel13452-bib-0072] Horvath, S., Oshima, J., Martin, G. M., Lu, A. T., Quach, A., Cohen, H., Felton, S., Matsuyama, M., Lowe, D., Kabacik, S., Wilson, J. G., Reiner, A. P., Maierhofer, A., Flunkert, J., Aviv, A., Hou, L., Baccarelli, A. A., Li, Y., Stewart, J. D., … Raj, K. (2018). Epigenetic clock for skin and blood cells applied to Hutchinson Gilford Progeria Syndrome and ex vivo studies. Aging, 10(7), 1758–1775. 10.18632/aging.101508 30048243 PMC6075434

[acel13452-bib-0073] Horvath, S., Pirazzini, C., Bacalini, M. G., Gentilini, D., Di Blasio, A. M., Delledonne, M., Mari, D., Arosio, B., Monti, D., Passarino, G., De Rango, F., D'Aquila, P., Giuliani, C., Marasco, E., Collino, S., Descombes, P., Garagnani, P., & Franceschi, C. (2015b). Decreased epigenetic age of PBMCs from Italian semi‐supercentenarians and their offspring. Aging, 7(12), 1159–1170. 10.18632/aging.100861 26678252 PMC4712339

[acel13452-bib-0074] Horvath, S., & Raj, K. (2018). DNA methylation‐based biomarkers and the epigenetic clock theory of ageing. Nature Reviews Genetics, 19(6), 371–384. 10.1038/s41576-018-0004-3 29643443

[acel13452-bib-0064] Horvath, S., Singh, K., Raj, K., Khairnar, S., Sanghavi, A., Shrivastava, A., Zoller, J. A., Li, C. Z., Herenu, C. B., Canatelli‐Mallat, M., Lehmann, M., Solberg Woods, L. C., Martinez, A. G., Wang, T., Chiavellini, P., Levine, A. J., Chen, H., Goya, R. G., & Katcher, H. L. (2020). Reversing age: Dual species measurement of epigenetic age with a single clock. bioRxiv, 2020.05.07.082917.

[acel13452-bib-0063] Horvath, S., Zoller, J. A., Haghani, A., Janinska, A. J., Raj, K., Breeze, C. E., Ernst, J., & Mattison, J. A. (2020). Epigenetic clock and methylation studies in the rhesus macaque. bioRxiv, 2020.09.21.307108. 10.1007/s11357-021-00429-8 PMC8599607 34487267

[acel13452-bib-0061] Horvath, S., Zoller, J. A., Haghani, A., Lu, A. T., Raj, K., Jasinska, A. J., Mattison, J. A., & Salmon, A. B. (2020). DNA methylation age analysis of rapamycin in common marmosets. bioRxiv, 2020.11.21.392779. 10.1007/s11357-021-00438-7 PMC8599537 34482522

[acel13452-bib-0075] Huh, C. J., Zhang, B., Victor, M. B., Dahiya, S., Batista, L. F. Z., Horvath, S., & Yoo, A. S. (2016). Maintenance of age in human neurons generated by microRNA‐based neuronal conversion of fibroblasts. eLife, 5(e18648), 1–14. 10.7554/eLife.18648 PMC5067114 27644593

[acel13452-bib-0076] Ichiyama, K., Chen, T., Wang, X., Yan, X., Kim, B.‐S., Tanaka, S., Ndiaye‐Lobry, D., Deng, Y., Zou, Y., Zheng, P., Tian, Q., Aifantis, I., Wei, L., & Dong, C. (2015). The methylcytosine dioxygenase Tet2 promotes DNA demethylation and activation of cytokine gene expression in T cells. Immunity, 42(4), 613–626. 10.1016/j.immuni.2015.03.005 25862091 PMC4956728

[acel13452-bib-0077] Ignjatovic, V., Lai, C., Summerhayes, R., Mathesius, U., Tawfilis, S., Perugini, M. A., & Monagle, P. (2011). Age‐related differences in plasma proteins: How plasma proteins change from neonates to adults. PLoS One, 6(2), e17213. 10.1371/journal.pone.0017213 21365000 PMC3041803

[acel13452-bib-0078] Ito, H., Udono, T., Hirata, S., & Inoue‐Murayama, M. (2018). Estimation of chimpanzee age based on DNA methylation. Scientific Reports, 8(1), 9998. 10.1038/s41598-018-28318-9 29968770 PMC6030051

[acel13452-bib-0079] Jasinska, A. J., Haghani, A., Zoller, J. A., Li, C. Z., Arenson, A., Ernst, J., Kavanagh, K., Jorgensen, M. J., Mattison, J. A., Wojta, K., Choi, O., DeYoung, J., Li, X., Rao, A. W., Coppola, G., Freimer, N. B., Woods, R. P., & Horvath, S. (2020). Epigenetic clock and methylation studies in vervet monkeys. bioRxiv, 2020.09.09.289801. 10.1007/s11357-021-00466-3 PMC9135907 34591235

[acel13452-bib-0080] Jia, K., Chen, D. I., & Riddle, D. L. (2004). The TOR pathway inter‐ acts with the insulin signaling pathway to regulate *C. elegans* larval development, metabolism and life span. Development, 131(16), 3897–3906. 15253933 10.1242/dev.01255

[acel13452-bib-0081] Jin, C., Lu, Y., Jelinek, J., Liang, S., Estecio, M. R. H., Barton, M. C., & Issa, J.‐p J. (2014). TET1 is a maintenance DNA demethylase that prevents methylation spreading in differentiated cells. Nucleic Acids Research, 42(11), 6956–6971. 10.1093/nar/gku372 24875481 PMC4066785

[acel13452-bib-0082] Johnson, S. C., Rabinovitch, P. S., & Kaeberlein, M. (2013). MTOR is a key modulator of ageing and age‐related disease. Nature, 493(7432), 338–345. 10.1038/nature11861 23325216 PMC3687363

[acel13452-bib-0083] Jung, M., & Pfeifer, G. P. (2015). Aging and DNA methylation. BMC Biology, 13(1), 7. 10.1186/s12915-015-0118-4 25637097 PMC4311512

[acel13452-bib-0084] Jung, S.‐E., Lim, S. M., Hong, S. R., Lee, E. H., Shin, K.‐J., & Lee, H. Y. (2019). DNA methylation of the ELOVL2, FHL2, KLF14, C1orf132/MIR29B2C, and TRIM59 genes for age prediction from blood, saliva, and buccal swab samples. Forensic Science International: Genetics, 38, 1–8. 30300865 10.1016/j.fsigen.2018.09.010

[acel13452-bib-0085] Jylhävä, J., Pedersen, N. L., & Hägg, S. (2017). Biological age predictors. EBioMedicine, 21, 29–36. 10.1016/j.ebiom.2017.03.046 28396265 PMC5514388

[acel13452-bib-0086] Kabacik, S., Horvath, S., Cohen, H., & Raj, K. (2018). Epigenetic ageing is distinct from senescence‐mediated ageing and is not prevented by telomerase expression. Aging, 10(10), 2800–2815. 10.18632/aging.101588 30332397 PMC6224244

[acel13452-bib-0087] Kapahi, P., Zid, B. M., Harper, T., Koslover, D., Sapin, V., & Benzer, S. (2004). Regulation of lifespan in Drosophila by modulation of genes in the TOR signaling pathway. Current Biology, 14(10), 885–890. 10.1016/j.cub.2004.03.059 15186745 PMC2754830

[acel13452-bib-0088] Katrinli, S., Stevens, J., Wani, A. H., Lori, A., Kilaru, V., van Rooij, S. J. H., Hinrichs, R., Powers, A., Gillespie, C. F., Michopoulos, V., Gautam, A., Jett, M., Hammamieh, R., Yang, R., Wildman, D., Qu, A., Koenen, K., Aiello, A. E., Jovanovic, T., … Smith, A. K. (2020). Evaluating the impact of trauma and PTSD on epigenetic prediction of lifespan and neural integrity. Neuropsychopharmacology, 1–8. 10.1038/s41386-020-0700-5 PMC7421899 32380512

[acel13452-bib-0089] Kennedy, B. K., Austriaco, N. R., Zhang, J., & Guarente, L. (1995). Mutation in the silencing gene S/R4 can delay aging in *S. cerevisiae* . Cell, 80(3), 485–496. 10.1016/0092-8674(95)90499-9 7859289

[acel13452-bib-0090] Klass, M. R. (1977). Aging in the nematode *Caenorhabditis elegans*: Major biological and environmental factors influencing life span. Mechanisms of Ageing and Development, 6, 413–429. 10.1016/0047-6374(77)90043-4 926867

[acel13452-bib-0091] Koch, C. M., Joussen, S., Schellenberg, A., Lin, Q., Zenke, M., & Wagner, W. (2012). Monitoring of cellular senescence by DNA‐methylation at specific CpG sites. Aging Cell, 11(2), 366–369. 10.1111/j.1474-9726.2011.00784.x 22221451

[acel13452-bib-0092] Koch, C. M., & Wagner, W. (2011). Epigenetic‐aging‐signature to determine age in different tissues. Aging, 3(10), 1018–1027. 22067257 10.18632/aging.100395 PMC3229965

[acel13452-bib-0093] Kordowitzki, P., Haghani, A., Zoller, J. A., Li, C. Z., Raj, K., Spangler, M. L., & Horvath, S. (2021). Epigenetic clock and methylation study of oocytes from a bovine model of reproductive aging. Aging Cell, 20(5). 10.1111/acel.13349 PMC8135012 33797841

[acel13452-bib-0094] Kozlenkov, A., Jaffe, A., Timashpolsky, A., Apontes, P., Rudchenko, S., Barbu, M., Byne, W., Hurd, Y., Horvath, S., & Dracheva, S. (2017). DNA methylation profiling of human prefrontal cortex neurons in heroin users shows significant difference between genomic contexts of hyper‐ and hypomethylation and a younger epigenetic age. Genes, 8(6), 152. 10.3390/genes8060152 PMC5485516 28556790

[acel13452-bib-0095] Kurotaki, N., Imaizumi, K., Harada, N., Masuno, M., Kondoh, T., Nagai, T., Ohashi, H., Naritomi, K., Tsukahara, M., Makita, Y., Sugimoto, T., Sonoda, T., Hasegawa, T., Chinen, Y., Tomita, H.‐A., Kinoshita, A., Mizuguchi, T., Yoshiura, K.‐I., Ohta, T., … Matsumoto, N. (2002). Haploinsufficiency of NSD1 causes Sotos syndrome. Nature Genetics, 30(4), 365–366. 10.1038/ng863 11896389

[acel13452-bib-0096] Lau, P. Y., & Fung, W. K. (2020). Evaluation of marker selection methods and statistical models for chronological age prediction based on DNA methylation. Legal Medicine, 47, 101744. 10.1016/j.legalmed.2020.101744 32659707

[acel13452-bib-0097] Lehallier, B., Gate, D., Schaum, N., Nanasi, T., Lee, S. E., Yousef, H., Moran Losada, P., Berdnik, D., Keller, A., Verghese, J., Sathyan, S., Franceschi, C., Milman, S., Barzilai, N., & Wyss‐Coray, T. (2019). Undulating changes in human plasma proteome profiles across the lifespan. Nature Medicine, 25(12), 1843–1850. 10.1038/s41591-019-0673-2 PMC7062043 31806903

[acel13452-bib-0098] Lemaître, J. F., Rey, B., Gaillard, J. M., Régis, C., Gilot, E., Pellerin, M., Haghani, A., Zoller, J. A., Li, C. Z., & Horvath, S. (2020). Epigenetic clock and DNA methylation studies of roe deer in the wild. bioRxiv, 2020.09.21.306613.

[acel13452-bib-0099] Levine, M. E. (2013). Modeling the rate of senescence: can estimated biological age predict mortality more accurately than chronological age? Journals of Gerontology Series A: Biomedical Sciences and Medical Sciences, 68(6), 667–674. 10.1093/gerona/gls233 PMC3660119 23213031

[acel13452-bib-0101] Levine, M. E., Lu, A. T., Bennett, D. A., & Horvath, S. (2015). Epigenetic age of the pre‐frontal cortex is associated with neuritic plaques, amyloid load, and Alzheimer’s disease related cognitive functioning. Aging, 7(12), 1198–1211. 10.18632/aging.100864 26684672 PMC4712342

[acel13452-bib-0100] Levine, M., McDevitt, R. A., Meer, M., Perdue, K., Di Francesco, A., Meade, T., Farrell, C., Thrush, K., Wang, M., Dunn, C., Pellegrini, M., de Cabo, R., & Ferrucci, L. (2020). A rat epigenetic clock recapitulates phenotypic aging and co‐localizes with heterochromatin. eLife, 9(e59201), 1–19. 10.7554/elife.59201 PMC7661040 33179594

[acel13452-bib-0102] Levine, M. E., Lu, A. T., Quach, A., Chen, B. H., Assimes, T. L., Bandinelli, S., Hou, L., Baccarelli, A. A., Stewart, J. D., Li, Y., Whitsel, E. A., Wilson, J. G., Reiner, A. P., Aviv, A., Lohman, K., Liu, Y., Ferrucci, L., & Horvath, S. (2018). An epigenetic biomarker of aging for lifespan and healthspan. Aging, 10(4), 573–591. 10.18632/aging.101414 29676998 PMC5940111

[acel13452-bib-0103] Levy, J. J., Titus, A. J., Petersen, C. L., Chen, Y., Salas, L. A., & Christensen, B. C. (2020). MethylNet: An automated and modular deep learning approach for DNA methylation analysis. BMC Bioinformatics, 21(1), 108. 10.1186/s12859-020-3443-8 32183722 PMC7076991

[acel13452-bib-0104] Li, C. Z., Haghani, A., Robeck, T. R., Villar, D., Lu, A. T., Zhang, J., Faulkes, C. G., Vu, H., Ablaeva, J., Adams, D. M., Ardehali, R., Arneson, A., Baker, C. S., Belov, K., Blumstein, D. T., Bors, E. K., Breeze, R. T., Brooke, J. L., … Mammalian Methylation Consortium (2021). Epigenetic predictors of maximum lifespan and other life history traits in mammals. bioRxiv, 2021.05.16.444078.

[acel13452-bib-0105] Li, X., Ploner, A., Wang, Y., Magnusson, P. K. E., Reynolds, C., Finkel, D., Pedersen, N. L., Jylhävä, J., & Hägg, S. (2020). Longitudinal trajectories, correlations and mortality associations of nine biological ages across 20‐years follow‐up. eLife, 9(E51507), 1–20. 10.7554/eLife.51507 PMC7012595 32041686

[acel13452-bib-0106] Lin, Q., Weidner, C. I., Costa, I. G., Marioni, R. E., Ferreira, M. R. P., Deary, I. J., & Wagner, W. (2016). DNA methylation levels at individual age‐associated CpG sites can be indicative for life expectancy. Aging, 8(2), 394–401. 10.18632/aging.100908 26928272 PMC4789590

[acel13452-bib-0107] Lindsey, J., McGill, N. I., Lindsey, L. A., Green, D. K., & Cooke, H. J. (1991). In vivo loss of telomeric repeats with age in humans. Mutation Research DNAging, 256(1), 45–48. 10.1016/0921-8734(91)90032-7 1944386

[acel13452-bib-0108] Little, T., O’Toole, A., Rambaut, A., Chandra, T., Marioni, R., & Pedersen, A. (2020). Methylation‐based age estimation in a wild mouse. bioRxiv, 2020.07.16.203687.

[acel13452-bib-0109] López‐Otín, C., Blasco, M. A., Partridge, L., Serrano, M., & Kroemer, G. (2013). The hallmarks of aging. Cell, 153(6), 1194. 10.1016/j.cell.2013.05.039 23746838 PMC3836174

[acel13452-bib-0110] Lowe, D., Horvath, S., & Raj, K. (2016). Epigenetic clock analyses of cellular senescence and ageing. Oncotarget, 7(8), 8524–8531. 10.18632/oncotarget.7383 26885756 PMC4890984

[acel13452-bib-0111] Lowe, R., Danson, A. F., Rakyan, V. K., Yildizoglu, S., Saldmann, F., Viltard, M., Friedlander, G., & Faulkes, C. G. (2020). DNA methylation clocks as a predictor for ageing and age estimation in naked mole‐rats, *Heterocephalus glaber* . Aging, 12(5), 4394–4406. 10.18632/aging.102892 32126024 PMC7093186

[acel13452-bib-0118] Lu, Y., Brommer, B., Tian, X., Krishnan, A., Meer, M., Wang, C., Vera, D. L., Zeng, Q., Yu, D., Bonkowski, M. S., Yang, J.‐H., Zhou, S., Hoffmann, E. M., Karg, M. M., Schultz, M. B., Kane, A. E., Davidsohn, N., Korobkina, E., Chwalek, K., … Sinclair, D. A. (2020). Reprogramming to recover youthful epigenetic information and restore vision. Nature, 588(7836), 124–129. 33268865 10.1038/s41586-020-2975-4 PMC7752134

[acel13452-bib-0114] Lu, A. T., Fei, Z., Haghani, A., Robeck, T. R., Zoller, J. A., Li, C. Z., Zhang, J., Ablaeva, J., Adams, D. M., Almunia, J., Ardehali, R., Arneson, A., Baker, C. S., Belov, K., Black, P., Blumstein, D. T., Bors, E. K., Breeze, C. E., Brooke, R. T., … Mammalian Methylation Consortium (2021). Universal DNA methylation age across mammalian tissues. bioRxiv, 2021.01.18.426733.

[acel13452-bib-0113] Lu, A. T., Hannon, E., Levine, M. E., Crimmins, E. M., Lunnon, K., Mill, J., Geschwind, D. H., & Horvath, S. (2017). Genetic architecture of epigenetic and neuronal ageing rates in human brain regions. Nature Communications, 8(1), 1–14. 10.1038/ncomms15353 PMC5454371 28516910

[acel13452-bib-0112] Lu, A. T., Hannon, E., Levine, M. E., Hao, K., Crimmins, E. M., Lunnon, K., Kozlenkov, A., Mill, J., Dracheva, S., & Horvath, S. (2016). Genetic variants near MLST8 and DHX57 affect the epigenetic age of the cerebellum. Nature Communications, 7(1), 1–9. 10.1038/ncomms10561 PMC4740877 26830004

[acel13452-bib-0115] Lu, A. T., Quach, A., Wilson, J. G., Reiner, A. P., Aviv, A., Raj, K., Hou, L., Baccarelli, A. A., Li, Y., Stewart, J. D., Whitsel, E. A., Assimes, T. L., Ferrucci, L., & Horvath, S. (2019a). DNA methylation GrimAge strongly predicts lifespan and healthspan. Aging, 11, 303–327. 30669119 10.18632/aging.101684 PMC6366976

[acel13452-bib-0116] Lu, A. T., Seeboth, A., Tsai, P.‐C., Sun, D., Quach, A., Reiner, A. P., Kooperberg, C., Ferrucci, L., Hou, L., Baccarelli, A. A., Li, Y., Harris, S. E., Corley, J., Taylor, A., Deary, I. J., Stewart, J. D., Whitsel, E. A., Assimes, T. L., Chen, W., … Horvath, S. (2019b). DNA methylation‐based estimator of telomere length. Aging, 11(16), 5895–5923. 10.18632/aging.102173 31422385 PMC6738410

[acel13452-bib-0117] Lu, A. T., Xue, L., Salfati, E. L., Chen, B. H., Ferrucci, L., Levy, D., Joehanes, R., Murabito, J. M., Kiel, D. P., Tsai, P.‐C., Yet, I., Bell, J. T., Mangino, M., Tanaka, T., McRae, A. F., Marioni, R. E., Visscher, P. M., Wray, N. R., Deary, I. J., … Horvath, S. (2018). GWAS of epigenetic aging rates in blood reveals a critical role for TERT. Nature Communications, 9(1), 387. 10.1038/s41467-017-02697-5 PMC5786029 29374233

[acel13452-bib-0119] Lunnon, K., Hannon, E., Smith, R. G., Dempster, E., Wong, C., Burrage, J., Troakes, C., Al‐Sarraj, S., Kepa, A., Schalkwyk, L., & Mill, J. (2016). Variation in 5‐hydroxymethylcytosine across human cortex and cerebellum. Genome Biology, 17(1), 27. 26883014 10.1186/s13059-016-0871-x PMC4756397

[acel13452-bib-0120] Maierhofer, A., Flunkert, J., Oshima, J., Martin, G. M., Haaf, T., & Horvath, S. (2017). Accelerated epigenetic aging in Werner syndrome. Aging, 9(4), 1143–1152. 10.18632/aging.101217 28377537 PMC5425119

[acel13452-bib-0121] Manukyan, M., & Singh, P. B. (2012). Epigenetic rejuvenation. Genes to Cells, 17(5), 337–343. 22487104 10.1111/j.1365-2443.2012.01595.x PMC3444684

[acel13452-bib-0122] Manukyan, M., & Singh, P. B. (2014). Epigenome rejuvenation: HP1*β* mobility as a measure of pluripotent and senescent chromatin ground states. Scientific Reports, 4(1), 4789. 24763337 10.1038/srep04789 PMC3999444

[acel13452-bib-0123] Marioni, R. E., Harris, S. E., Shah, S., McRae, A. F., von Zglinicki, T., Martin‐Ruiz, C., Wray, N. R., Visscher, P. M., & Deary, I. J. (2016). The epigenetic clock and telomere length are independently associated with chronological age and mortality. International Journal of Epidemiology, 45(2), 424–432. 10.1093/ije/dyw041 27075770 PMC4864882

[acel13452-bib-0124] Marioni, R. E., Shah, S., McRae, A. F., Chen, B. H., Colicino, E., Harris, S. E., Gibson, J., Henders, A. K., Redmond, P., Cox, S. R., Pattie, A., Corley, J., Murphy, L., Martin, N. G., Montgomery, G. W., Feinberg, A. P., Fallin, M. D., Multhaup, M. L., Jaffe, A. E., … Deary, I. J. (2015a). DNA methylation age of blood predicts all‐cause mortality in later life. Genome Biology, 16(1), 25. 10.1186/s13059-015-0584-6 25633388 PMC4350614

[acel13452-bib-0125] Marioni, R. E., Shah, S., McRae, A. F., Ritchie, S. J., Muniz‐Terrera, G., Harris, S. E., Gibson, J., Redmond, P., Cox, S. R., Pattie, A., Corley, J., Taylor, A., Murphy, L., Starr, J. M., Horvath, S., Visscher, P. M., Wray, N. R., & Deary, I. J. (2015b). The epigenetic clock is correlated with physical and cognitive fitness in the Lothian Birth Cohort 1936. International Journal of Epidemiology, 44(4), 1388–1396. 25617346 10.1093/ije/dyu277 PMC4588858

[acel13452-bib-0126] Marioni, R. E., Suderman, M., Chen, B. H., Horvath, S., Bandinelli, S., Morris, T., Beck, S., Ferrucci, L., Pedersen, N. L., Relton, C. L., Deary, I. J., & Hägg, S. (2019). Tracking the epigenetic clock across the human life course: A meta‐analysis of longitudinal cohort data. The Journals of Gerontology: Series A, 74(1), 57–61. 10.1093/gerona/gly060 PMC6298183 29718110

[acel13452-bib-0127] Martin‐Herranz, D. E., Aref‐Eshghi, E., Bonder, M. J., Stubbs, T. M., Choufani, S., Weksberg, R., Stegle, O., Sadikovic, B., Reik, W., & Thornton, J. M. (2019). Screening for genes that accelerate the epigenetic aging clock in humans reveals a role for the H3K36 methyltransferase NSD1. Genome Biology, 20(1). 10.1186/s13059-019-1753-9 PMC6693144 31409373

[acel13452-bib-0128] Mayer, W., Niveleau, A., Walter, J., Fundele, R., & Haaf, T. (2000). Demethylation of the zygotic paternal genome. Nature, 403(6769), 501–502. 10.1038/35000656 10676950

[acel13452-bib-0129] Mayne, B., Korbie, D., Kenchington, L., Ezzy, B., Berry, O., & Jarman, S. (2020). A DNA methylation age predictor for zebrafish. Aging, 12, 24817–24835. 10.18632/aging.202400 33353889 PMC7803548

[acel13452-bib-0131] McCartney, D. L., Hillary, R. F., Stevenson, A. J., Ritchie, S. J., Walker, R. M., Zhang, Q., Morris, S. W., Bermingham, M. L., Campbell, A., Murray, A. D., Whalley, H. C., Gale, C. R., Porteous, D. J., Haley, C. S., McRae, A. F., Wray, N. R., Visscher, P. M., McIntosh, A. M., Evans, K. L., … Marioni, R. E. (2018). Epigenetic prediction of complex traits and death. Genome Biology, 19(1), 136. 10.1186/s13059-018-1514-1 30257690 PMC6158884

[acel13452-bib-0130] McCartney, D. L., Zhang, F., Hillary, R. F., Zhang, Q., Stevenson, A. J., Walker, R. M., Bermingham, M. L., Boutin, T., Morris, S. W., Campbell, A., Murray, A. D., Whal‐ley, H. C., Porteous, D. J., Hayward, C., Evans, K. L., Chandra, T., Deary, I. J., McIntosh, A. M., Yang, J. … Marioni, R. E. (2019). An epigenome‐wide association study of sex‐ specific chronological ageing. Genome Medicine, 12(1), 1–11. 31892350 10.1186/s13073-019-0693-z PMC6938636

[acel13452-bib-0132] McCrory, C., Fiorito, G., Hernandez, B., Polidoro, S., O’Halloran, A. M., Hever, A., Ni Cheallaigh, C., Lu, A. T., Horvath, S., Vineis, P., & Kenny, R. A. (2021). GrimAge outperforms other epigenetic clocks in the prediction of age‐related clinical phenotypes and all‐cause mortality. The Journals of Gerontology: Series A, 76(5), 741–749. 10.1093/gerona/glaa286 PMC8087266 33211845

[acel13452-bib-0133] McEwen, L. M., O’Donnell, K. J., McGill, M. G., Edgar, R. D., Jones, M. J., MacIsaac, J. L., Lin, D. T. S., Ramadori, K., Morin, A., Gladish, N., Garg, E., Unternaehrer, E., Pokhvisneva, I., Karnani, N., Kee, M. Z. L., Klengel, T., Adler, N. E., Barr, R. G., Letourneau, N., … Kobor, M. S. (2020). The PedBE clock accurately estimates DNA methylation age in pediatric buccal cells. Proceedings of the National Academy of Sciences, 117(38), 23329–23335. 10.1073/pnas.1820843116 PMC7519312 31611402

[acel13452-bib-0134] Meer, M. V., Podolskiy, D. I., Tyshkovskiy, A., & Gladyshev, V. N. (2018). A whole lifespan mouse multi‐tissue DNA methylation clock. eLife, 7(e.40675), 1–16. 10.7554/elife.40675 PMC6287945 30427307

[acel13452-bib-0135] Murphy, C. T., McCarroll, S. A., Bargmann, C. I., Fraser, A., Kamath, R. S., Ahringer, J., Li, H., & Kenyon, C. (2003). Genes that act downstream of DAF‐16 to influence the lifespan of Caenorhabditis elegans. Nature, 424(6946), 277–284. 10.1038/nature01789 12845331

[acel13452-bib-0136] Nilsson, E., Jansson, P. A., Perfilyev, A., Volkov, P., Pedersen, M., Svensson, M. K., Poulsen, P., Ribel‐Madsen, R., Pedersen, N. L., Almgren, P., Fadista, J., Ronn, T., Klarlund Pedersen, B., Scheele, C., Vaag, A., & Ling, C. (2014). Altered DNA methylation and differential expression of genes influencing metabolism and inflammation in adipose tissue from subjects with type 2 diabetes. Diabetes, 63(9), 2962–2976. 10.2337/db13-1459 24812430

[acel13452-bib-0137] Ocampo, A., Reddy, P., Martinez‐Redondo, P., Platero‐Luengo, A., Hatanaka, F., Hishida, T., Li, M., Lam, D., Kurita, M., Beyret, E., Araoka, T., Vazquez‐Ferrer, E., Donoso, D., Roman, J. L., Xu, J., Rodriguez Esteban, C., Nuñez, G., Nuñez Delicado, E., Campistol, J. M., … Izpisua Belmonte, J. C. (2016). In vivo amelioration of age‐associated hallmarks by partial reprogramming. Cell, 167(7), 1719–1733. 27984723 10.1016/j.cell.2016.11.052 PMC5679279

[acel13452-bib-0139] Olova, N., Krueger, F., Andrews, S., Oxley, D., Berrens, R. V., Branco, M. R., & Reik, W. (2018). Comparison of whole‐genome bisulfite sequencing library preparation strategies identifies sources of biases affecting DNA methylation data. Genome Biology, 19(1), 33. 10.1186/s13059-018-1408-2 29544553 PMC5856372

[acel13452-bib-0138] Olova, N., Simpson, D. J., Marioni, R. E., & Chandra, T. (2019). Partial reprogramming induces a steady decline in epigenetic age before loss of somatic identity. Aging Cell, 18(1), 1–7. 10.1111/acel.12877 PMC6351826 30450724

[acel13452-bib-0140] Pearson, K. J., Baur, J. A., Lewis, K. N., Peshkin, L., Price, N. L., Labinskyy, N., Swindell, W. R., Kamara, D., Minor, R. K., Perez, E., Jamieson, H. A., Zhang, Y., Dunn, S. R., Sharma, K., Pleshko, N., Woollett, L. A., Csiszar, A., Ikeno, Y., Le Couteur, D., … de Cabo, R. (2008). Resveratrol delays age‐related deterioration and mimics transcriptional aspects of dietary restriction without extending life span. Cell Metabolism, 8(2), 157–168. 10.1016/j.cmet.2008.06.011 18599363 PMC2538685

[acel13452-bib-0141] Perna, L., Zhang, Y., Mons, U., Holleczek, B., Saum, K.‐U., & Brenner, H. (2016). Epigenetic age acceleration predicts cancer, cardiovascular, and all‐cause mortality in a German case cohort. Clinical Epigenetics, 8(1), 64. 10.1186/s13148-016-0228-z 27274774 PMC4891876

[acel13452-bib-0142] Peters, M. J., Joehanes, R., Pilling, L. C., Schurmann, C., Conneely, K. N., Powell, J., Reinmaa, E., Sutphin, G. L., Zhernakova, A., Schramm, K., Wilson, Y. A., Kobes, S., Tukiainen, T., Ramos, Y. F., Göring, H. H. H., Fornage, M., Liu, Y., Gharib, S. A., Stranger, B. E., … Johnson, A. D. (2015). The transcriptional landscape of age in human peripheral blood. Nature Communications, 6(1), 1–14. 10.1038/ncomms9570 PMC4639797 26490707

[acel13452-bib-0143] Petkovich, D. A., Podolskiy, D. I., Lobanov, A. V., Lee, S.‐G., Miller, R. A., & Gladyshev, V. N. (2017). Using DNA methylation profiling to evaluate biological age and longevity interventions. Cell Metabolism, 25(4), 954–960. 10.1016/j.cmet.2017.03.016 28380383 PMC5578459

[acel13452-bib-0144] Phillip, J. M., Wu, P.‐H., Gilkes, D. M., Williams, W., McGovern, S., Daya, J., Chen, J., Aifuwa, I., Lee, J. S. H., Fan, R., Walston, J., & Wirtz, D. (2017). Biophysical and biomolecular determination of cellular age in humans. Nature Biomedical Engineering, 1, 7. 10.1038/s41551-017-0093 PMC6675017 31372309

[acel13452-bib-0145] Pinho, G. M., Martin, J. G. A., Farrell, C., Haghani, A., Zoller, J. A., Zhang, J., Snir, S., Pellegrini, M., Wayne, R. K., Blumstein, D. T., & Horvath, S. (2021). Hibernation slows epigenetic aging in yellow‐bellied marmots. bioRxiv, 2021.03.07.434299. 10.1038/s41559-022-01679-1 PMC8986532 35256811

[acel13452-bib-0146] Polanowski, A. M., Robbins, J., Chandler, D., & Jarman, S. N. (2014). Epigenetic estimation of age in humpback whales. Molecular Ecology Resources, 14(5), 976–987. 10.1111/1755-0998.12247 24606053 PMC4314680

[acel13452-bib-0147] Prado, N. A., Brown, J. L., Zoller, J. A., Haghani, A., Yao, M., Bagryanova, L. R., Campana, M. G., Maldonado, J. E., Raj, K., Schmitt, D., Robeck, T. R., & Horvath, S. (2020). Epigenetic clock and methylation studies in elephants. bioRxiv, 2020.09.22.308882. 10.1111/acel.13414 PMC8282242 34118182

[acel13452-bib-0148] Quach, A., Levine, M. E., Tanaka, T., Lu, A. T., Chen, B. H., Ferrucci, L., Ritz, B., Bandinelli, S., Neuhouser, M. L., Beasley, J. M., Snetselaar, L., Wallace, R. B., Tsao, P. S., Absher, D., Assimes, T. L., Stewart, J. D., Li, Y., Hou, L., Baccarelli, A. A., … Horvath, S. (2017). Epigenetic clock analysis of diet, exercise, education, and lifestyle factors. Aging, 9(2), 419–446. 10.18632/aging.101168 28198702 PMC5361673

[acel13452-bib-0149] Raj, K., Szladovits, K., Haghani, A., Zoller, J. A., Li, C. Z., & Horvath, S. (2020). Epigenetic clock and methylation studies in cats. bioRxiv. 10.1007/s11357-021-00445-8 PMC8599556 34463900

[acel13452-bib-0150] Ridker, P. M., Buring, J. E., Cook, N. R., & Rifai, N. (2003). C‐reactive protein, the metabolic syndrome, and risk of incident cardiovascular events: An 8‐year follow‐up of 14 719 initially healthy American women. Circulation. 10.1161/01.CIR.0000055014.62083.05 12551861

[acel13452-bib-0151] Robertson, N. A., Hillary, R. F., McCartney, D. L., Terradas‐Terradas, M., Higham, J., Sproul, D., Deary, I. J., Kirschner, K., Marioni, R. E., & Chandra, T. (2019). Age‐related clonal haemopoiesis is associated with increased epigenetic age. Current Biology, 29(16), R786–R787. 31430471 10.1016/j.cub.2019.07.011

[acel13452-bib-0152] Ross, K. M., Carroll, J. E., Horvath, S., Hobel, C. J., Coussons‐Read, M. E., & Schetter, C. D. (2020). Epigenetic age and pregnancy outcomes: GrimAge acceleration is associated with shorter gestational length and lower birthweight. Clinical Epigenetics, 12(120), 1–11. 10.1186/s13148-020-00909-2 PMC7409637 32762768

[acel13452-bib-0153] Sailer, L. L., Haghani, A., Zoller, J. A., Li, C. Z., Ophir, A. G., & Horvath, S. (2020). Pair bonding slows epigenetic aging and alters methylation in brains of prairie voles. bioRxiv. 10.1038/s41598-024-67641-2 PMC11286801 39075111

[acel13452-bib-0154] Sarkar, T. J., Quarta, M., Mukherjee, S., Colville, A., Paine, P., Doan, L., Tran, C. M., Chu, C. R., Horvath, S., Qi, L. S., Bhutani, N., Rando, T. A., & Sebastiano, V. (2020). Transient non‐integrative expression of nuclear reprogramming factors promotes multifaceted amelioration of aging in human cells. Nature Communications, 11(1), 1–12. 10.1038/s41467-020-15174-3 PMC7093390 32210226

[acel13452-bib-0155] Schachtschneider, K. M., Schook, L. B., Meudt, J. J., Shanmuganayagam, D., Zoller, J. A., Haghani, A., Li, C. Z., Zhang, J., Yang, A., Raj, K., & Horvath, S. (2020). Epigenetic clock and DNA methylation analysis of porcine models of aging and obesity. bioRxiv, 2020.09.29.319509. 10.1007/s11357-021-00439-6 PMC8599541 34523051

[acel13452-bib-0156] Simpkin, A. J., Hemani, G., Suderman, M., Gaunt, T. R., Lyttleton, O., Mcardle, W. L., Ring, S. M., Sharp, G. C., Tilling, K., Horvath, S., Kunze, S., Peters, A., Walden‐berger, M., Ward‐Caviness, C., Nohr, E. A., Sørensen, T. I., Relton, C. L., & Smith, G. D. (2016). Prenatal and early life influences on epigenetic age in children: A study of mother‐offspring pairs from two cohort studies. Human Molecular Genetics, 25(1), 191–201. 26546615 10.1093/hmg/ddv456 PMC4690495

[acel13452-bib-0157] Simpkin, A. J., Howe, L. D., Tilling, K., Gaunt, T. R., Lyttleton, O., McArdle, W. L., Ring, S. M., Horvath, S., Smith, G. D., & Relton, C. L. (2017). The epigenetic clock and physical development during childhood and adolescence: Longitudinal analysis from a UK birth cohort. International Journal of Epidemiology, 46(2), 549–558. 10.1093/ije/dyw307 28089957 PMC5722033

[acel13452-bib-0158] Singh, P. B., & Zacouto, F. (2010). Nuclear reprogramming and epigenetic rejuvenation. Journal of Biosciences, 35(2), 315–319. 20689186 10.1007/s12038-010-0034-2

[acel13452-bib-0159] Slieker, R. C., Relton, C. L., Gaunt, T. R., Slagboom, P. E., & Heijmans, B. T. (2018). Age‐related DNA methylation changes are tissue‐specific with ELOVL2 promoter methylation as exception. Epigenetics and Chromatin, 11(1), 25. 10.1186/s13072-018-0191-3 29848354 PMC5975493

[acel13452-bib-0160] Snir, S., Farrell, C., & Pellegrini, M. (2019). Human epigenetic ageing is logarithmic with time across the entire lifespan. Epigenetics, 14(9), 912–926. 10.1080/15592294.2019.1623634 31138013 PMC6691990

[acel13452-bib-0161] Snir, S., VonHoldt, B. M., & Pellegrini, M. (2016). A statistical frame‐work to identify deviation from time linearity in epigenetic aging. PLoS Computational Biology, 12(11), 1005183. 10.1371/journal.pcbi.1005183 PMC5106012 27835646

[acel13452-bib-0162] Starnawska, A., Tan, Q., Soerensen, M., McGue, M., Mors, O., Børglum, A. D., Christensen, K., Nyegaard, M., & Christiansen, L. (2019). Epigenome‐wide association study of depression symptomatology in elderly monozygotic twins. Translational Psychiatry, 9(1), 1–14. 31477683 10.1038/s41398-019-0548-9 PMC6718679

[acel13452-bib-0163] Stubbs, T. M., Bonder, M. J., Stark, A. K., Krueger, F., von Meyenn, F., Stegle, O., & Reik, W. (2017). Multi‐tissue DNA methylation age predictor in mouse. Genome Biology, 18(1), 68. 28399939 10.1186/s13059-017-1203-5 PMC5389178

[acel13452-bib-0164] Sugrue, V. J., Zoller, J. A., Narayan, P., Lu A. T., Ortega‐Recalde, O. J., Grant, M. J., Bawden, C. S., Rudiger, S. R., Haghani, A., Bond, D. M., Hore, R. R., Garratt, M., Sears, K. E., Wang, N., Yang, X. W., Snell, R. G., Hore, T. A., & Horvath S. (2021). Castration delays epigenetic aging and feminizes DNA methylation at androgen‐regulated loci. eLife, 10(e64932), 1–20. 10.7554/elife.64932 PMC8260231 34227937

[acel13452-bib-0165] Tanaka, T., Biancotto, A., Moaddel, R., Moore, A. Z., Gonzalez‐Freire, M., Aon, M. A., Candia, J., Zhang, P., Cheung, F., Fantoni, G., Semba, R. D., & Ferrucci, L. (2018). Plasma proteomic signature of age in healthy humans. Aging Cell, 17(5), e12799. 10.1111/acel.12799 29992704 PMC6156492

[acel13452-bib-0166] Tatar, M., Kopelman, A., Epstein, D., Tu, M. P., Yin, C. M., & Garofalo, R. S. (2001). A mutant Drosophila insulin receptor homolog that extends life‐span and impairs neuroendocrine function. Science, 292(5514), 107–110. 10.1126/science.1057987 11292875

[acel13452-bib-0167] Teschendorff, A. E., Yang, Z., Wong, A., Pipinikas, C. P., Jiao, Y., Jones, A., Anjum, S., Hardy, R., Salvesen, H. B., Thirlwell, C., Janes, S. M., Kuh, D., & Widschwendter, M. (2015). Correlation of smoking‐associated DNA methylation changes in buccal cells with DNA methylation changes in epithelial cancer. JAMA Oncology, 1(4), 476–485. 10.1001/jamaoncol.2015.1053 26181258

[acel13452-bib-0168] Thermo Fisher , (2020). “SNaPshot™ Multiplex Kit”. https://www.thermofisher.com/order/catalog/product/4323163#/4323163

[acel13452-bib-0169] Thompson, M. J., Chwiałkowska, K., Rubbi, L., Lusis, A. J., Davis, R. C., Srivastava, A., Korstanje, R., Churchill, G. A., Horvath, S., & Pellegrini, M. (2018). A multi‐tissue full lifespan epigenetic clock for mice. Aging, 10(10), 2832–2854. 10.18632/aging.101590 30348905 PMC6224226

[acel13452-bib-0170] Thompson, M. J., vonHoldt, B., Horvath, S., & Pellegrini, M. (2017). An epigenetic aging clock for dogs and wolves. Aging, 9(3), 1055–1068. 10.18632/aging.101211 28373601 PMC5391218

[acel13452-bib-0171] Tibshirani, R. (1997). The lasso method for variable selection in the cox model. Statistics in Medicine, 16(4), 385–395. 10.1002/(sici)1097-0258(19970228)16:4<385::aid-sim380>3.0.co;2-3 9044528

[acel13452-bib-0172] Tissenbaum, H. A., & Ruvkun, G. (1998). An insulin‐like signaling pathway affects both longevity and reproduction in Caenorhabditis elegans. Genetics, 148(2), 703–717. 10.1093/genetics/148.2.703 9504918 PMC1459840

[acel13452-bib-0173] Travers, M. E., Mackay, D. J. G., Dekker Nitert, M., Morris, A. P., Lindgren, C. M., Berry, A., Johnson, P. R., Hanley, N., Groop, L. C., McCarthy, M. I., & Gloyn, A. L. (2013). Insights into the molecular mechanism for type 2 diabetes susceptibility at the KCNQ1 locus from temporal changes in imprinting status in human islets. Diabetes, 62(3), 987–992. 10.2337/db12-0819 23139357 PMC3581222

[acel13452-bib-0174] Vellai, T., Takacs‐Vellai, K., Zhang, Y., Kovacs, A. L., Orosz, L., & Müller, F. (2003). Influence of TOR kinase on lifespan in *C. elegans* . Nature, 426(6967), 620–620. 10.1038/426620a 14668850

[acel13452-bib-0175] Vetter, V. M., Spira, D., Banszerus, V. L., & Demuth, I. (2020). Epigenetic clock and leukocyte telomere length are associated with vitamin D status, but not with functional assessments and frailty in the berlin aging study II. The Journals of Gerontology: Series A. 10.1093/gerona/glaa101 32324874

[acel13452-bib-0176] Walker, R. F., Liu, J. S., Peters, B. A., Ritz, B. R., Wu, T., Ophoff, R. A., & Horvath, S. (2015). Epigenetic age analysis of children who seem to evade aging. Aging, 7(5), 334–339. 10.18632/aging.100744 25991677 PMC4468314

[acel13452-bib-0177] Wallace, S. S. (2014). Base excision repair: A critical player in many games. DNA Repair, 19, 14–26. 10.1016/j.dnarep.2014.03.030 24780558 PMC4100245

[acel13452-bib-0178] Wang, T., Ma, J., Hogan, A. N., Fong, S., Licon, K., Tsui, B., Kreisberg, J. F., Adams, P. D., Carvunis, A.‐R., Bannasch, D. L., Ostrander, E. A., & Ideker, T. (2020). Quantitative translation of dog‐to‐human aging by conserved remodeling of the DNA methylome. Cell Systems, 11(2), 176–185. 10.1016/j.cels.2020.06.006 32619550 PMC7484147

[acel13452-bib-0179] Wang, T., Tsui, B., Kreisberg, J. F., Robertson, N. A., Gross, A. M., Yu, M. K., Carter, H., Brown‐Borg, H. M., Adams, P. D., & Ideker, T. (2017). Epigenetic aging signatures in mice livers are slowed by dwarfism, calorie restriction and rapamycin treatment. Genome Biology, 18(1), 57. 10.1186/s13059-017-1186-2 28351423 PMC5371228

[acel13452-bib-0180] Weidner, C., Lin, Q., Koch, C., Eisele, L., Beier, F., Ziegler, P., Bauerschlag, D., Jöckel, K.‐H., Erbel, R., Mühleisen, T., Zenke, M., Brümmendorf, T., & Wagner, W. (2014). Aging of blood can be tracked by DNA methylation changes at just three CpG sites. Genome Biology, 15(2), R24. 10.1186/gb-2014-15-2-r24 24490752 PMC4053864

[acel13452-bib-0181] Weindruch, R., Walford, R. L., & Guthrie, D. (1986). The retardation of aging in mice by dietary restriction: Longevity, cancer, immunity and lifetime energy intake. Journal of Nutrition, 116(4), 641–6541. 10.1093/jn/116.4.641 3958810

[acel13452-bib-0182] Wilkinson, G. S., Adams, D. M., Haghani, A., Lu, A. T., Zoller, J., Breeze, C. E., Arnold, B. D., Ball, H. C., Carter, G. G., Cooper, L. N., Dechmann, D. K., Devanna, P., Fasel, N. J., Galazyuk, A. V., Günther, L., Hurme, E., Jones, G., Knörnschild, M., Lattenkamp, E. Z., … Horvath, S. (2021). DNA methylation predicts age and provides insight into exceptional longevity of bats. Nature Communications, 12(1), 1–13. 10.1038/s41467-021-21900-2 PMC7955057 33712580

[acel13452-bib-0183] Wolf, E. J., Logue, M. W., Hayes, J. P., Sadeh, N., Schichman, S. A., Stone, A., Salat, D. H., Milberg, W., McGlinchey, R., & Miller, M. W. (2016). Accelerated DNA methylation age: Associations with PTSD and neural integrity. Psychoneuroendocrinology, 63, 155–162. 10.1016/j.psyneuen.2015.09.020 26447678 PMC4695261

[acel13452-bib-0184] Wolffe, A. P., Jones, P. L., & Wade, P. A. (1999). DNA demethylation. Proceedings of the National Academy of Sciences of the United States of America, 96, 5894–5896 10339513 10.1073/pnas.96.11.5894 PMC34201

[acel13452-bib-0185] Wu, X., Huang, Q., Javed, R., Zhong, J., Gao, H., & Liang, H. (2019). Effect of tobacco smoking on the epigenetic age of human respiratory organs. Clinical Epigenetics, 11(1), 183. 10.1186/s13148-019-0777-z 31801625 PMC6894291

[acel13452-bib-0186] Xu C., Qu, H., Wang, G., Xie, B., Shi, Y., Yang, Y., Zhao, Z., Hu, L., Fang, X., Yan, J., & Feng, L. (2015). A novel strategy for forensic age prediction by DNA methylation and support vector regression model. Scientific Reports, 5(1), 1–10. 10.1038/srep17788 PMC4669521 26635134

[acel13452-bib-0187] Yang, R., Wu, G. W. Y., Verhoeven, J. E., Gautam, A., Reus, V. I., Kang, J. I., Flory, J. D., Abu‐Amara, D., Hood, L., Doyle, F. J., Yehuda, R., Marmar, C. R., Jett, M., Hammamieh, R., Mellon, S. H., & Wolkowitz O. M. (2020). A DNA methylation clock associated with age‐related illnesses and mortality is accelerated in men with combat PTSD. Molecular Psychiatry, 1–11. 10.1038/s41380-020-0755-z 32382136

[acel13452-bib-0188] Yang, Z., Wong, A., Kuh, D., Paul, D. S., Rakyan, V. K., Leslie, R. D., Zheng, S. C., Widschwendter, M., Beck, S., & Teschendorff, A. E. (2016). Correlation of an epigenetic mitotic clock with cancer risk. Genome Biology, 17(1), 205. 10.1186/s13059-016-1064-3 27716309 PMC5046977

[acel13452-bib-0189] Yin, Y., Morgunova, E., Jolma, A., Kaasinen, E., Sahu, B., Khund‐Sayeed, S., Das, P. K., Kivioja, T., Dave, K., Zhong, F., Nitta, K. R., Taipale, M., Popov, A., Ginno, P. A., Domcke, S., Yan, J., Schübeler, D., Vinson, C., & Taipale, J. (2017). Impact of cytosine methylation on DNA binding specificities of human transcription factors. Science, 356, 6337. 10.1126/science.aaj2239 PMC8009048 28473536

[acel13452-bib-0190] Yu, M., Hazelton, W. D., Luebeck, G. E., & Grady, W. M. (2020). Epigenetic aging: More than just a clock when it comes to cancer. Cancer Research, 80(3), 367–374. 10.1158/0008-5472.CAN-19-0924 31694907 PMC7002254

[acel13452-bib-0191] Zbieć‐Piekarska, R., Spólnicka, M., Kupiec, T., Parys‐Proszek, A., Makowska, Ż., Pałeczka, A., Kucharczyk, K., Płoski, R., & Branicki, W. (2015). Development of a forensically useful age prediction method based on DNA methylation analysis. Forensic Science International: Genetics, 17, 173–179. 26026729 10.1016/j.fsigen.2015.05.001

[acel13452-bib-0195] Zhang, Y., Hapala, J., Brenner, H., & Wagner, W. (2017). Individual CpG sites that are associated with age and life expectancy become hypomethylated upon aging. Clinical Epigenetics, 9(1), 1–6. 10.1186/s13148-017-0315-9 28168006 PMC5288846

[acel13452-bib-0192] Zhang, J. M., Kamath, G. M., & Tse, D. N. (2019). Valid post‐clustering differential analysis for single‐cell RNA‐Seq. bioRxiv, 463265. 10.1016/j.cels.2019.07.012 PMC7202736 31521605

[acel13452-bib-0194] Zhang, Q., Vallerga, C. L., Walker, R. M., Lin, T., Henders, A. K., Montgomery, G. W., He, J. I., Fan, D., Fowdar, J., Kennedy, M., Pitcher, T., Pearson, J., Halliday, G., Kwok, J. B., Hickie, I., Lewis, S., Anderson, T., Silburn, P. A., Mellick, G. D., … Visscher, P. M. (2019). Improved precision of epigenetic clock estimates across tissues and its implication for biological ageing. Genome Medicine, 11(1), 54. 10.1186/s13073-019-0667-1 31443728 PMC6708158

[acel13452-bib-0196] Zhang, Y., Wilson, R., Heiss, J., Breitling, L. P., Saum, K.‐U., Schöttker, B., Holleczek, B., Waldenberger, M., Peters, A., & Brenner, H. (2017). DNA methylation signatures in peripheral blood strongly predict all‐cause mortality. Nature Communications, 8, 14617. 10.1038/ncomms14617 PMC5357865 28303888

[acel13452-bib-0193] Zhang, J., Yang, C., Wang, C., Liu, D., Lao, G., Liang, Y., Sun, K., Luo, H., Tan, Q., Ren, M., & Yan, L. I. (2016). AGE‐induced keratinocyte MMP‐9 expression is linked to TET2‐mediated CpG demethylation. Wound Repair and Regeneration, 24(3), 489–500. 10.1111/wrr.12426 26913994

[acel13452-bib-0197] Zheng, S. C., Widschwendter, M., & Teschendorff, A. E. (2016). Epigenetic drift, epigenetic clocks and cancer risk. Epigenomics, 8(5), 705–719. 27104983 10.2217/epi-2015-0017

[acel13452-bib-0198] Zou, H., & Hastie, T. (2005). Regularization and variable selection via the elastic net. Journal of the Royal Statistical Society: Series B (Statistical Methodology), 67(2), 301–320.

